# D-2-HG Inhibits *IDH1^mut^* Glioma Growth via FTO Inhibition and Resultant m6A Hypermethylation

**DOI:** 10.1158/2767-9764.CRC-23-0271

**Published:** 2024-03-22

**Authors:** Sean T. Pianka, Tie Li, Terry J. Prins, Blaine S.C. Eldred, Bryan M. Kevan, Haowen Liang, Serendipity Zapanta Rinonos, Harley I. Kornblum, David A. Nathanson, Matteo Pellegrini, Linda M. Liau, Phioanh Leia Nghiemphu, Timothy F. Cloughesy, Albert Lai

**Affiliations:** 1 Department of Neurology, UCLA Medical Center, Los Angeles, California.; 2 Department of Neurosurgery, University of Florida, College of Medicine, Gainesville, Florida.; 3 Department of Molecular and Medical Pharmacology, UCLA, Los Angeles, California.; 4 Department of Molecular, Cell and Developmental Biology, UCLA, Los Angeles, California.; 5 Department of Neurosurgery, UCLA Medical Center, Los Angeles, California.

## Abstract

*IDH1^mut^* gliomas produce high levels of D-2-hydroxyglutarate (D-2-HG), an oncometabolite capable of inhibiting α-ketoglutarate–dependent dioxygenases critical to a range of cellular functions involved in gliomagenesis. *IDH1^mut^* gliomas also exhibit slower growth rates and improved treatment sensitivity compared with their *IDH1^wt^* counterparts. This study explores the mechanism driving apparent reduced growth in *IDH1^mut^* gliomas. Specifically, we investigated the relationship between *IDH1^mut^* and the RNA N^6^-methyladenosine (m6A) demethylases FTO and ALKBH5, and their potential for therapeutic targeting. We investigated the role of D-2-HG and m6A in tumor proliferation/viability using glioma patient tumor samples, patient-derived gliomaspheres, and U87 cells, as well as with mouse intracranial *IDH1^wt^* gliomasphere xenografts. Methylation RNA immunoprecipitation sequencing (MeRIP-seq) RNA sequencing was used to identify m6A-enriched transcripts in *IDH1^mut^* glioma. We show that IDH1^mut^ production of D-2-HG is capable of reducing glioma cell growth via inhibition of the m6A epitranscriptomic regulator, FTO, with resultant m6A hypermethylation of a set of mRNA transcripts. On the basis of unbiased MeRIP-seq epitranscriptomic profiling, we identify *ATF5* as a hypermethylated, downregulated transcript that potentially contributes to increased apoptosis. We further demonstrate how targeting this pathway genetically and pharmacologically reduces the proliferative potential of malignant *IDH1^wt^* gliomas, both *in vitro* and *in vivo*. Our work provides evidence that selective inhibition of the m6A epitranscriptomic regulator FTO attenuates growth in *IDH1^wt^* glioma, recapitulating the clinically favorable growth phenotype seen in the *IDH1^mut^* subtype.

**Significance:**

We show that IDH1^mut^-generated D-2-HG can reduce glioma growth via inhibition of the m6A demethylase, FTO. FTO inhibition represents a potential therapeutic target for *IDH1^wt^* gliomas and possibly in conjunction with IDH1^mut^ inhibitors for the treatment of *IDH1^mut^* glioma. Future studies are necessary to demonstrate the role of ATF5 downregulation in the indolent phenotype of *IDH1^mut^* gliomas, as well as to identify other involved gene transcripts deregulated by m6A hypermethylation.

## Introduction

Glioma is the most common primary brain cancer in adults with approximately 20,000 patients diagnosed yearly in the United States (1). Extensive genetic and molecular characterization of patient gliomas have established several glioma subtypes (2, 3). One major subtype, defined by a heterozygous mutation in *isocitrate dehydrogenase (IDH)* 1 or 2, comprises 35% of all adult diffuse gliomas (including ∼80% of World Health Organization grade II–III gliomas and 12% of all glioblastomas; refs. 4, 5). The *IDH* mutation represents a driver mutation of gliomagenesis but also appears to mediate favorable clinical attributes such as slower tumor growth and increased overall survival (4–7).

Mechanistically, IDH1 loses the ability to convert isocitrate to α-ketoglutarate (α-KG) when IDH1^mut^ and IDH1^wt^ proteins heterodimerize, and instead gains the ability to convert α-KG to D-2-hydroxyglutarate (D-2-HG; ref. 8). This can lead to a paradoxical change in α-KG levels (9). However, the major influence of the *IDH* mutation results from supraphysiologic levels of intracellular and extracellular D-2-HG that act as a competitive inhibitor of a wide variety of Fe(II)-dependent and α-KG–dependent dioxygenases in glioma cells and their microenvironment (10, 11). How D-2-HG may both initiate gliomas and slow growth remains to be understood.

Recently, the ability of D-2-HG to inhibit α-KG–dependent dioxygenases has intersected with the rapidly evolving field of epitranscriptomics, specifically regarding N^6^-methyladenosine (m6A), the most common modification in eukaryotic mRNA. Current evidence indicates that m6A modifications are dynamically regulated through m6A methyltransferases (“writers”; refs. 12, 13) and demethylases (“erasers”; refs. 14–17) to impact mRNA splicing, secondary structure, export from the nucleus, intracellular localization, transcript stability, translation efficiency, miRNA processing, and RNA–protein interactions (18–22). The mammalian m6A erasers identified to date, fat mass and obesity-associated protein (FTO, also known as ALKBH9) and AlkB homolog 5 (ALKBH5; refs. 16, 17), have been suggested to have oncogenic roles in different cancer types including leukemia, glioma, breast cancer, and lung cancer (23–27). FTO can bind many RNA species and can demethylate various substrates not limited to m6A (28). Recent studies also posit that D-2-HG treatment can inhibit glioma cell viability in a dose-dependent fashion (Supplementary Data, Su and colleagues*,* 2018) and that D-2-HG can inhibit FTO in *IDH1^mut^* acute myeloid leukemia cells to increase m6A abundance and slow cell proliferation (25, 26). However, differential m6A methylation in glioma is poorly understood and warrants further investigation (29), because many studies ignore *IDH1* mutation status, yield conflicting results (30, 31), or do not account for potential upstream pathways that may be influencing m6A methylation and tumor growth (27, 32, 33).

The focus of this study is to confirm the growth suppressive ability of D-2-HG in *IDH1^mut^* gliomas and determine whether this could be mediated by m6A dysregulation. Combining epitranscriptomic techniques applied to patient glioma samples and gliomaspheres, we demonstrate the presence of RNA m6A hypermethylation in *IDH1^mut^* gliomas that can be directly induced via D-2-HG–mediated inhibition of FTO demethylase (erasure) activity. We also show how this *IDH1^m^^ut^* → D-2-HG ⊣ FTO axis may inhibit tumor growth through downregulation of activating transcription factor 5 (*ATF5*) mRNA, a transcription factor involved in proliferation and apoptosis in glioma (34, 35). These findings highlight a potential biological mechanism underlying the favorable prognosis that *IDH1^mut^* confers upon glioma patients and provide evidence that FTO is a viable therapeutic target in *IDH1^wt^* glioblastomas.

## Materials and Methods

Refer to Supplementary Materials and Methods for detailed information on Immunoblotting, Intracellular D-2-HG Quantification, Gene-specific m6A qRT-PCR, The Cancer Genome Atlas (TCGA) Gene Expression Analyses, Gliomasphere Microarray Analyses, Animal Procedures, *Gaussia* Luciferase Tumor Burden Estimation, and Survival Analysis.

### Patient Tissue Sample Acquisition

Clinically annotated *IDH1^mut^* (*n* = 44) and *IDH1^wt^* (*n* = 36) fresh frozen patient glioma tumors were acquired through the University of California Los Angeles (UCLA) Brain Tumor Translational Resource, and under approval of the UCLA Institutional Review Board in accordance with the U.S. Common Rule (Supplementary Table S1). Each sample was subjected to histologic quality controls confirming the presence of ≥70% tumor versus normal brain tissue, and *IDH1* status was established using IDH1^mut^ IHC and Sanger sequencing.

### Cell Culture and Treatments

*IDH1^mut^* expression was induced in previously established U87 cells using *IDH1^R132H^*-pLPCX and *IDH1^wt^*-pLPCX retroviral constructs, with blank-pLPCX transduced lines acting as vector controls (36–38). IDH1^mut^ expression was confirmed in all cell lines with immunoblotting. Immortalized normal human astrocytes (NHA; ref. 39) with stabilized expression of hTERT, E6, and E7 were provided by Dr. Russell Pieper (University of California, San Francisco, CA; 2016). *IDH1^mut^* (*n* = 4) and *IDH1^wt^* (*n* = 19) patient-derived gliomaspheres were provided by Dr. Harley Kornblum (University of California, Los Angeles, CA; collected between 2013 and 2023; ref. 40) and Dr. David Nathanson (University of California, Los Angeles, CA; collected between 2013 and 2023; Supplementary Table S2). BT142 hemizygous *IDH1^mut/^^−^* gliomaspheres were obtained from ATCC (ATCC, catalog no. ATS-1018, RRID:CVCL_X501, 2013). U87 (ATCC, catalog no. HTB-14, RRID:CVCL_0022, 2007), HEK293T (ATCC, catalog no. CRL-3216, RRID:CVCL_0063, 2007), and NHA cell lines were maintained in DMEM cell culture medium supplemented with 10% FBS and penicillin/streptomycin, cultured at 37°C and 5% CO_2_. Gliomaspheres were maintained in DMEM/F12 supplemented with B27, heparin, EGF, and bFGF. Previously expanded cell lines were thawed and passaged one additional time before being used in the described experiments. Neither *Mycoplasma* testing nor authentication was performed. Octyl-D-2-HG (Cayman Chemical, catalog nos. 16374 and 21123) treatment dosages were selected on the basis of previous work to achieve intracellular concentrations of D-2-HG levels seen in *IDH1^mut^* glioma cells (41). Two IDH1^mut^ inhibitors were utilized in our study: C35 (Xcess Biosciences, Inc. catalog no. M60068-2s) and AG881 (Selleck Chemicals, catalog no. S8611). In addition, we tested two FTO inhibitors: FB23-2 (MedChemExpress, catalog no. HY-127103) and meclofenamic acid (MA; Cayman Chemicals, catalog no. 70550). All pharmacologic treatment agents were dissolved in DMSO (Sigma-Aldrich, catalog no. 472301), and cells were treated with indicated concentrations and time courses. Equivalent quantities of DMSO (or PBS in D-2-HG experiments) were used as treatment control conditions across all described experiments.

### Total RNA and mRNA Isolation

Total RNA was purified from cellular samples using RNeasy Mini Kits (Qiagen, catalog no. 74104). For methylation RNA immunoprecipitation sequencing (MeRIP-seq) experiment, total RNA was isolated using TRIzol Reagent (Ambion, catalog no. 15596018). PolyATtract mRNA Isolation Systems Kit (Promega, catalog no. Z5300) was used to purify mRNA from total RNA samples.

### m6A RNA Dot Blot

Total RNA and poly A^+^ purified mRNA from samples were subjected to m6A dot blots using m6A-specific antibody (Synaptic Systems, catalog no. 202003, RRID:AB_2279214). Briefly, mRNA was isolated from total RNA using Dynabeads mRNA Purification Kits (Thermo Fisher Scientific, catalog no. 61006) per the manufacturer's instructions. At least 20 µg of total RNA was used for each sample. Purified mRNA was diluted to a concentration of 2 ng/µL, and then heated at 70°C for 10 minutes to disrupt secondary RNA structures. A total of 2 µL of mRNA was then added directly to Hybond-N+ membrane optimized for nucleic acid transfer, followed by 15 minutes of UV-light exposure to induce cross-linking. Membranes were washed in TBS with 0.05% Tween 20 (TBST) wash buffer for three 10-minute rounds. Membranes were then incubated in blocking buffer (TBST w/5% milk and 2.0% BSA) for 1 hour at room temperature, followed by incubation with anti-m6A antibody in a 1:2,000 dilution in fresh blocking buffer overnight at 4°C. Three rounds of 10 minutes TBST washes were then followed by incubation with goat anti-rabbit IgG-HRP (horseradish peroxidase; 1:5,000 dilution; Abcam, catalog no. ab6721, RRID:AB_955447) in blocking buffer for 1 hour at room temperature. After an additional three rounds of 10 minutes TBST washes, membranes processed using Pierce ECL Western Blotting Substrate kit (Thermo Fisher Scientific, catalog no. 32109) was used for HRP signal visualization. ChemiDoc XRS+ Imaging system (RRID: SCR_019690) and image lab software (Bio-Rad, catalog no. 1709690, RRID:SCR_014210) was used to capture chemiluminescent images of immunoblot membranes.

### Quantification of m6A

Quantification of m6A in total RNA was achieved using EpiQuik m6A RNA Methylation Quantification Kits (Colorimetric; EpiGenTek, catalog no. P900548) according to the manufacturer's directions. Briefly, 200 ng of purified RNA was added to the provided strip-well plate frame, followed by incubation at 37°C for 90 minutes. Capture antibodies for m6A were then added to the solution and incubated for 60 minutes at room temperature, followed by the addition of corresponding detection antibodies and 30 minutes of incubation at room temperature. After the addition of enhancer solution and several rounds of washing per the manufacturer's protocol, detection solution was added followed by 10 minutes of incubation at room temperature away from light. A stop solution was utilized to halt the enzymatic reaction and the 450 nm absorbance immediately read on a Wallac Victor2 microplate reader (Perkin Elmer, catalog no. 1420-012). Relative m6A content is proportional to measured OD intensities for each sample and were calculated using the following formula: 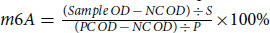 (where NC OD = 450 nm absorbance for negative m6A control RNA, PC OD = 50 nm absorbance for positive m6A control RNA, S = ng of sample RNA, P = ng of PC RNA). ANOVA or two-tailed Student *t* tests were used to compare means between groups, with statistical significance set at *P* ≤ 0.05.

### IDH1^mut^ Forced Expression into Gliomaspheres

Lentiviruses were produced from pUltra-*IDH1^mut^-EGFP* or blank pUltra-*EGFP* plasmids (Addgene, catalog no. 24129, RRID:Addgene_24129) packaged with pMD2.G VSV-G envelope plasmid (Addgene, catalog no. 12259, RRID:Addgene_12259) and pCMVR8.74 packaging plasmid (Addgene, catalog no. 22036, RRID:Addgene_22036) in HEK293T cells cultured in regular DMEM/10% FBS. Plasmids were transfected into HEK293T using X-tremeGENE HP DNA Transfection Reagent (Sigma-Aldrich, catalog no. XTGHP-RO). HEK293T cells were allowed to incubate for 48 hours, at which time the lentivirus-containing media was harvested, aliquoted, and stored at −80°C. Target glioma cells were disassociated to the single-cell level and seeded in gliomasphere-compatible media (absent penicillin/streptomycin). Cells were transduced with lentivirus-containing media and regular culture media in a 1:5 ratio. 1 µg/mL of polybrene was added to facilitate transduction efficiency. Cells remained in lentivirus-containing media for no more than 48 hours, at which point media was replaced with standard culture media. After 12–15 days, all cells exhibited 100% GFP positivity indicating successful transduction. Sequencing and Western blot analyses were performed for confirmation.

### FTO, ALKBH5, and ATF5 Knockdown and Overexpression

For knockdown experiments, plasmids for piLenti-*FTO-shRNA-GFP* (5′-TTTCTCACACTGCACAAGCATGGCTGCTT-3′), piLenti*-ALKBH5-shRNA-GFP* (5′-CGTGTCCGTGTCCTTCTTTAGCGACTCTG-3′), piLenti-*ATF5-A-siRNA-GFP* (5′-GGGCTGGGATGGCTCGTAGACTATGGGAA-3′), piLenti-*ATF5-B-siRNA-GFP* (5′-TTGGATACTCTGGACTTGCTGGCCATCTA-3′), or piLenti-*NSC-shRNA-GFP* (5′-AACAGGCACACGTCCCAGCGT-3′) were constructed on a piLenti-*U6-shRNA-GFP-Puro* vector backbone (GenTarget, Inc. catalog no. SH-U6-GP). Lentiviruses containing these constructs were packaged as described above. For overexpression, lentiviruses containing *FTO^wt^* (pMIRNA1-Flag-*FTO^wt^*) and *FTO^mut^* (pMIRNA1-Flag-*FTO^mut^*) were also produced in this manner. FTO^mut^ contains point mutations (H231A; D233A) that disrupt enzymatic activity to provide the control. Target sphere infections for knockdown and overexpression are same as above. Three days after infection, 1 µg/mL of puromycin was applied for 7 days to select for successfully transduced cells. GFP imaging was used to validate transduction efficiency, and Western blot analyses were used to confirm successful target manipulation.

### Quantification of Cell Viability or Proliferation

For adherent cell lines (U87, NHA), 3-(4,5-dimethylthiazol-2-yl)-2,5-diphenyltetrazolium bromide (MTT) assays were employed to test cell viability. Briefly, a uniform number of cells were cultured in 24-well plates under specified times and treatment durations, and then exposed to premixed MTT solution (0.5 mg/mL in regular culture media). Cells were incubated at 37°C for 1 hour. The MTT solution was then removed, and the MTT-reduced formazan product was extracted from the cells following lysing with 300 µL of DMSO. Formazan concentrations were measured at 560 nm absorbance using a Wallac Victor2 microplate reader and a background subtraction of 660 nm absorbance. Three independent experiments were performed for each experimental condition. Data were analyzed in GraphPad Prism 9 (RRID:SCR_002798) and results presented as a “mean±SEM.” For gliomaspheres, growth was determined using sphere volume measurement. Briefly, live images of gliomaspheres in culture were taken using a Leica DMi8-440 inverted fluorescent microscope. Sphere volumes in live images were then calculated using ImageJ (RRID:SCR_003070). A minimum of 100 spheres were imaged for each experimental condition, and volumes were averaged and analyzed in Prism 9 with results presented as a “mean±SEM” with a unit of mm^3^. Gliomaspheres in each experiment were grown for the same number of days, ranging between 9 and 15 days depending on the cell line. Unless otherwise stated, two-tailed Student *t* tests were used to compare means between groups, with statistical significance set at *P * ≤ 0.05.

### Drug Affinity Responsive Target Stability Assay

Drug affinity responsive target stability (DARTS) assay was performed as described previously (42). Briefly, total protein from U87 glioma cell lysate was used for DARTS assay, which contains FTO and ALKBH5 proteins. A total of 200 µg protein in 100 µL assay buffer was preincubated with D-2-HG as pointed concentration for 30 minutes. Then, they were equally divided into two aliquots: one with added protease (1 µg of protease: 100 µg of protein) and the other served as control. The protease digestion reaction was 15 minutes at room temperature. Potential D-2-HG interactions with protein components exert a protective effect against protease digestion. The protective effects were measured by Western blot analysis using anti-FTO (Novus, catalog no. NB110-60935, RRID:AB_925405), anti-ALKBH5 (Novus, catalog no. NBP1-82188, RRID:AB_11037354), and anti-α-tubulin (Sigma-Aldrich, catalog no. T6199, RRID:AB_477583; negative control) antibodies; quantitation was performed by densitometry.

### MeRIP-seq Differential Expression and m6A Methylation Analyses

A minimum of 400 µg of total RNA from patient tumor, gliomasphere, or glioma cell culture was subjected to poly A^+^ purification to isolate ≥10 µg of mRNA using the Dynabeads mRNA Purification kit (Thermo Fisher Scientific, catalog no. 61006). Transcripts were fragmented into approximately 100 nt segments as described previously (43). Dynabeads were coupled to m6A-specific antibodies (Synaptic Systems, catalog no. 202 003, RRID:AB_2279214) using the Dynabeads Antibody Coupling kit (Thermo Fisher Scientific, catalog no. 14311D). Washing, elution, extraction, and cleanup of m6A-enriched mRNA was performed per the manufacturer's protocols, described in further detail in the abovementioned publication. TruSeq Stranded mRNA Library Preparation kits (Illumina, catalog no. 20020594) were used for all sequencing library preparations, with unique adapter indices ligated to each sample to allow for multiplexing (TruSeq RNA Single Indices Set A, Illumina, catalog no. 20020492). Library quantification and quality control was performed using KAPA Library Quantification kits (Roche, catalog no. KK4828). For each sample, we performed high-throughput sequencing on an m6A-immunoprecipitated fraction (MeRIP-seq) as well as a mRNA-only fraction [RNA sequencing (RNA-seq)]. Sequencing was conducted at the UCLA Neuroscience Genomics Core on *Illumina* Hi-Seq4000 system. Each sample underwent 75 nt paired-end sequencing, giving a read depth of ≥30 million per multiplexed sample.

We conducted MeRIP-seq analysis using RADAR (44), a differential m6A analytic method developed in *R*, and the GRCh38 genome. Briefly, adapter sequences were trimmed using the FASTX-Toolkit (RRID:SCR_005534) and reads were filtered for mRNA transcripts using SortMeRNA (RRID:SCR_014402; ref. 45). Data were kept segregated by *IDH1* status (mutant vs. wildtype) and as m6A-immunoprecipitated mRNA or total mRNA. Reads were then mapped to the Gencode GRCh38 primary assembly with STAR (RRID:SCR_004463; ref. 46) using transcriptome mode to generate aligned BAM files for import into *R* Project for Statistical Computing (RRID:SCR_001905) and RADAR. GTF annotation files for this purpose were obtained through Gencode (RRID:SCR_014966). RADAR differential analysis was then conducted by subjecting our m6A-immunoprecipitated mRNA and total mRNA fractions to algorithmic m6A peak calling and annotation, comparing *IDH1^mut^* and *IDH1^wt^* cell lines using RADAR (44). Custom *R* scripts were written to intersect these results with TCGA (RRID:SCR_003193) RNA-seq data on the basis of strict criteria: (i) differential m6A expression detected by RADAR must have had a *P* < 0.01, (ii) a given transcript must be differentially expressed in TCGA data between *IDH^mut^* and *IDH^wt^* patients (absolute value log_2_ fold change >1.0; *P* < 1.0 × 10^−6^), and (iii) the differential m6A methylation must conform to m6A conventions (i.e., an increase in RADAR m6A inversely correlated with RNA expression in TCGA data).

### Apoptotic Activity Measurements

For quantification of changes in apoptotic activity in octyl-D-2-HG, FB23-2, and MA-treated cells, the Caspase-Glo 3/7 Assay Kit (Promega, catalog no. G8090) was used. The assay is a homogenous luminescent assay that captures Caspase 3 and Caspase 7 activities. This is achieved with a luminogenic substrate containing the tetrapeptide sequence DEVD. The substrate is cleaved by caspases, thus generating a luminescent luciferase signal proportional to the amount of caspase activity present in the samples. The manufacturer's protocol was followed in the execution of all experiments. Briefly, cells were cultured in white-walled 96-well plates and treated with the specified compounds in triplicate. A total of 100 µL of Caspase-Glo 3/7 Reagent was added to each well. Plates were placed on a shaker at 300 RPM for 30 seconds and incubated at room temperature for 3 hours. Luminescence for each sample was then measured on a Wallac Victor2 microplate reader.

For visualization of fluorescently-labeled apoptosis activity in octyl-D-2-HG, FB23-2, and MA-treated cells, the Apoptosis/Necrosis Assay kit (Abcam, catalog no. ab176749) was used, following the manufacturer's protocol. The assay stains phosphatidylserine exposed on the external leaflet of plasma membranes with a green fluorescent apopxin indicator (Ex/Em = 490/525 nm), while necrotic cells are labeled with a red fluorescent and membrane-impermeable 7-AAD (7-Aminoactinomycin D) indicator (Ex/Em = 550/650 nm) and healthy cells are visualized with a blue CytoCalcein Violet 450 indicator (Ex/Em = 405/450 nm). Briefly, cell lines were cultured in regular media in 24-well plates and treated with the specified compounds in triplicate. After 3 days, the plated cells were washed twice with 100 µL of provided Assay Buffer and resuspended in 200 µL of Assay Buffer with 2 µL of Apopxin Green Indicator (100X stock), 1 µL of 7-AAD Necrosis Indicator (200X stock), and 1 µL of CytoCalcein 450 Indicator (200X stock). Cells were incubated at room temperature for 60 minutes, followed by two 100 µL Assay Buffer washes. Cells were then imaged under a fluorescent microscope. Apoptotic cells were visualized using the FITC channel (Ex/Em = 490/525 nm). Necrotic cells were visualized using the Texas Red channel (Ex/Em = 550/650 nm). Healthy cells were visualized with a violet channel (Ex/Em = 405/450 nm).

### MazF-qRT-PCR

To further confirm *ATF5* m6A hypermethylation, we employed another m6A mRNA identification technique known as MazF-qRT-PCR. MazF is an ACA sequence–specific mRNA endoribonuclease found in *Escherichia coli*, and m6A modifications serve to protect RNA from MazF cleavage activity. MeRIP-seq called three m6A peaks in the final *ATF5*, among which only one motif (localized within the first m6A peak; 5′-TCAAGAAGGAGCTGGAmACAGATGGAAGmACTTCTTCCTAGATGCCCCGCCC-3′) was amenable to MazF-RT-PCR detection. In the 81 bases upstream and 114 bases downstream of the GAmACA motif, there are also two other ACA sites (non-m6A RRACA motifs), providing an approximately 195-base region suitable for qRT-PCR sequencing. We designed forward (5′-ACCTGACCTGGAAGCTATGG-3′) and reverse (5′-CAAAGGAGGGGAGGGACAG-3′) primers for this purpose. The remainder of the experiment proceeded according to the MazF methodology described in Imanishi and colleagues, 2017 (47).

### Animal Studies

All animal studies (see Supplementary Materials and Methods) were conducted under approval of the UCLA Institutional Animal Care and Use Committee.

### Data Availability Statement

All data generated in this study are available upon request from the corresponding author.

## Results

### Patient *IDH1^mut^* Gliomas and Gliomaspheres Exhibit Increased D-2-HG and m6A Content

**FIGURE 1:**
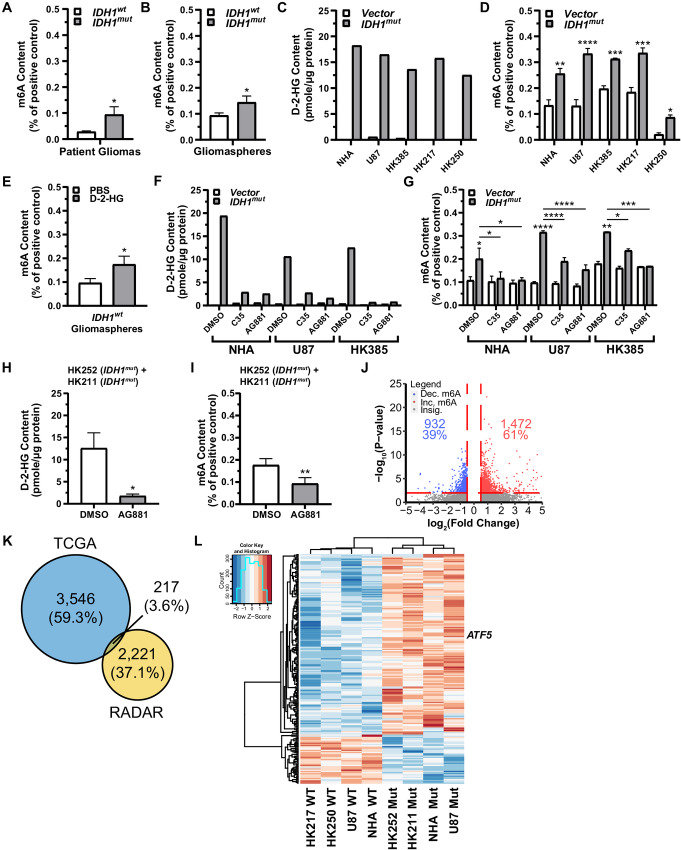
IDH1^mut^ production of D-2-HG induces RNA m6A hypermethylation in gliomas. **A,***IDH1^mut^* patient gliomas manifested RNA m6A hypermethylation, *IDH1^mut^* (*n* = 12) versus *IDH1^wt^* (*n* = 12; *P* ≤ 0.04). **B,***IDH1^mut^* gliomaspheres exhibited increased m6A content, *IDH1^mut^* (*n* = 4) versus *IDH1^wt^* (*n* = 7; *P* ≤ 0.04). In NHA lines, U87 cells, and *IDH1^wt^* gliomaspheres, *IDH1^mut^* forced expression elevates intracellular D-2-HG content (*n* = 1; **C**) and induce m6A enrichment [ANOVA, *F*(1,27) = 115.0, *P* ≤ 0.0001; asterisks indicate *post hoc* Newman–Keuls comparison with vector for each cell type] (**D**). **E,** Octyl-D-2-HG treatment (0.5 mmol/L) of *IDH1^wt^* gliomaspheres increased m6A content compared with PBS treatment controls (*n* = 6; paired *t* test; *P* ≤ 0.01). **F,** D-2-HG content in HK385, U87, and NHA cells following treatment with IDH1^mut^ inhibitors (C35 = 2 µmol/L; AG881 = 1 µmol/L), quantified using D-2-HG enzymatic assay. **G,** Effect of IDH1^mut^ inhibitor on m6A content in HK385, U87, and NHA cells with *IDH1^mut^* forced expression [ANOVA, IDH1^mut^ inhibitor treatment: *F*(8,32) = 12.9, *P* ≤ 0.0001; vector vs. *IDH1^mut:^ F*(1,32) = 79.22, *P* ≤ 0.0001; inhibitor and *IDH1* status interaction: *F*(8,32) = 7.3, *P* ≤ 0.0001; asterisks indicate *post hoc* Newman–Keuls comparison with vector or between the groups as indicated by horizontal bars]. Pooled data from native *IDH1^mut^* gliomaspheres (HK252, HK211) demonstrating a reduction in D-2-HG (one-tailed paired *t* test; *P* ≤ 0.04; **H**) and m6A content (paired *t* test; *P* ≤ 0.003; **I**) following IDH1^mut^ inhibitor AG881 treatment (1.0 µmol/L) in comparison with DMSO control. **J,** Volcano plot showing the RADAR log_2_ fold change in m6A expression against −log_10_ converted *P* values between *IDH1^mut^* and *IDH1^wt^* U87, NHA, and gliomasphere cells. Transcript sites with an absolute value log_2_ fold change of less than 0.5 are excluded (void region in between vertical, dashed red lines). The horizontal, dashed red line indicates the demarcation of RADAR *P* values <0.01. **K,** Venn diagram showing the total number of genes fitting TCGA criteria (TCGA *P* value <1.0 × 10^−6^; absolute value log_2_ fold change in RNA expression of 1.0 or greater between *IDH1^mut^* and *IDH1^wt^* patients) and RADAR criteria (*P <* 0.01) for all transcripts. Note that the total RADAR gene number is slightly larger here than the totals in **J**, because this list includes all *P* < 0.01 transcripts (including those with low magnitude fold changes). **L,** Supervised hierarchical clustering heat map of intersectional TCGA and RADAR data between U87, NHA, and gliomasphere cell lines that conformed to m6A and RNA expression conventions (i.e., an increase in RADAR m6A expression corresponded to a decrease in TCGA RNA expression). Heat map colors represent sample (columns) natural log converted normalized RADAR m6A values as a difference from the transcript (rows) average across genotype. A transcript of interest, *ATF5*, is identified. *, *P* ≤ 0.05; **, *P* ≤ 0.01; ***, *P* ≤ 0.001; and ****, *P* ≤ 0.0001 compared with relevant controls. Unless otherwise stated, *P* values indicate unpaired Student *t* test comparisons with the control, or between two groups as indicated by the horizontal line.

FTO and ALKBH5 are m6A erasers belonging to the superfamily of α-KG–dependent dioxygenases susceptible to D-2-HG inhibition (11, 48–50). We hypothesized that *IDH1^mut^* gliomas generating D-2-HG should therefore manifest a phenotype of global RNA m6A hypermethylation. We obtained *IDH1^mut^* (*n* = 12) and *IDH1^wt^*(*n* = 12) fresh frozen glioma samples from the UCLA Brain Tumor Translational Resource to examine their total RNA m6A levels. Details of pathologic diagnoses and patient cohort characteristics are summarized in Supplementary Table S1. As expected, m6A quantification using the colorimetric EpiQuik m6A RNA Methylation Quantification kit showed significantly higher m6A levels in *IDH1^mut^* gliomas versus *IDH1^wt^* gliomas (*P* ≤ 0.04; Fig. 1A). Although antibody-based approaches for m6A do have limitations including cross-reactivity to other RNA modifications, and limited utility for quantification of m6A stoichiometry, EpiQuik m6A testing has recently been confirmed to provide comparable results to mass spectrometry (51). Among *IDH1^mut^* (*n* = 4) and *IDH1^wt^* (*n* = 7) gliomaspheres assessed, all four *IDH1^mut^* gliomaspheres expressing the *IDH1^wt/R132H^* heterozygous genotype manifested higher levels of intracellular D-2-HG (*P* ≤ 0.003; Supplementary Fig. S1A) as expected and m6A content as measured by EpiQuik (*P* ≤ 0.04; Fig. 1B) and visualized by dot blot (Supplementary Fig. S1B). Notably, BT142 gliomaspheres with an uncommon hemizygous *IDH1^mut/^*^−^ genotype (thus unable to generate D-2-HG which requires the IDH1^mut^ and IDH1^wt^ protein heterodimer to convert α-KG to D-2-HG), demonstrate lower D-2-HG and m6A levels like that of *IDH1^wt^* lines (Supplementary Fig. S1A and S1B).

We next examined whether forcibly expressing *IDH1^mut^* in NHA, U87, or gliomasphere cell lines could induce m6A hypermethylation. Upon confirmation of increased intracellular D-2-HG in forced expressing *IDH1^mut^* lines compared with their respective vector controls (*n* = 1; Fig. 1C), we found that *IDH1^mut^* lines also demonstrated higher m6A levels in total RNA (Fig. 1D) and poly A^+^ purified mRNA isolates (Supplementary Fig. S1C). Three *IDH1^wt^* gliomasphere lines (HK385, HK217, and HK250) were also transfected with lentivirus vectors delivering pUltra-*IDH1^R132H^-EGFP* plasmid or an empty vector pUltra-*GFP* control. Upon confirmation that the three gliomasphere lines possessed increased intracellular D-2-HG content (*n* = 1; Fig. 1C), we observed m6A enrichment in each line after *IDH1^mut^* overexpression (Fig. 1D). Maintenance of gliomaspheres in differentiating media conditions (2% FBS) did not appear to affect m6A enrichment in *IDH1^mut^* gliomaspheres (Supplementary Fig. S1D).

As mentioned previously, dynamic regulation of mRNA m6A epitranscriptomic modifications depends on the balance between m6A writer and eraser activity. To address the possibility that differential expression of m6A writers and erasers contributes to the increased m6A content we observed in *IDH1^mut^* gliomas, we examined mRNA transcript levels of these genes in patient glioma tissue data in TCGA (RRID:SCR_003193) accessed via the GlioVis (RRID:SCR_023877) database portal and visualization tool and gliomasphere transcriptomic microarray data previously summarized in Laks and colleagues, 2016 (5, 52, 53). Microarray data showed similar expression of m6A writers and erasers among *IDH1^wt^* and *IDH1^mut^* gliomaspheres (Supplementary Fig. S1E), and TCGA data showed that *METTL3*, *METTL14*, *ALKBH5* show minimal magnitude differences between *IDH^mut^* and *IDH^wt^* patients, whereas *WTAP* and *FTO* (absolute value log_2_ fold change of >0.9) show larger differences. However, the direction of these changes, if functionally important, (e.g., increased *FTO* and decreased *WTAP* expression in the *IDH^mut^*) would result in decreased m6A content instead of the increased m6A that we observed (Supplementary Fig. S1F). Despite the limitations in this analysis given the differing baseline genetic backgrounds between *IDH1^wt^* and *IDH1^mut^* gliomas, these data indicate differences in writer and eraser expression is unlikely to explain m6A increases in *IDH1^mut^* gliomas. Taken together, these experiments indicate that *IDH1^mut^* gliomas are associated with increased levels of m6A hypermethylation and suggest a key role for D-2-HG.

### D-2-HG is Both Necessary and Sufficient to Induce m6A Hypermethylation

To investigate whether D-2-HG was sufficient to induce m6A hypermethylation, we treated multiple *IDH1^wt^* cells with cell membrane-permeable octyl-D-2-HG. Octyl-D-2-HG treatment yielded a noticeable increase in m6A abundance in gliomaspheres, as quantitated by EpiQuik (Fig. 1E) and visualized by dot blot in both gliomasphere (Supplementary Fig. S1G) and LN18, U87, and NHA cells lines (Supplementary Fig. S1H).

We next tested the effect of inhibiting D-2-HG production to determine whether D-2-HG is necessary to establish m6A hypermethylation in *IDH1^mut^* glioma. Employing two pharmacologic inhibitors of IDH1^mut^, AG881, and C35 (54, 55), we confirmed that 72 hour treatment with either C35 (2 µmol/L) or AG881 (1 µmol/L) reduced intracellular D-2-HG levels in *IDH1^mut^* forced expression lines (NHA, U87 cells, and HK385 gliomaspheres), with no change in D-2-HG content seen in C35- or AG881-treated *IDH1^wt^* vector controls (*n* = 1; Fig. 1F). As expected, treatment with IDH1^mut^ pharmacologic inhibitors reduced the abundance of m6A in these *IDH1^mut^*cells (Fig. 1G). Native *IDH1^mut^* gliomaspheres (HK252 and HK211) also exhibited reductions in D-2-HG and m6A upon treatment with AG881 (Fig. 1H and I). In the hemizygous *IDH1^mut/^^−^* BT142 gliomaspheres with nearly undetectable D-2-HG level comparable to that of *IDH1^wt^* glioma cells (Supplementary Fig. S1A), m6A abundance remained at pretreatment levels following AG881 and C35 exposure (Supplementary Fig. S1I). Western blot analysis confirmed no change in writer or eraser expression with D-2-HG (Supplementary Fig. S1J). Taken together, these experiments provide strong evidence that D-2-HG production is necessary for m6A hypermethylation in *IDH1^mut^* gliomas.

### *IDH1^mut^* Gliomas Harbor a Set of m6A Hypermethylated mRNAs That are Downregulated

We performed an unbiased screen of m6A-enriched transcripts across *IDH1^mut^* and *IDH1^wt^* U87 and NHA cell lines as well as four gliomasphere lines (*IDH1^mut^*: HK211, HK252; *IDH1^wt^*: HK217, HK250), using MeRIP-seq (56). To identify a set of differentially methylated and expressed transcripts, we then subjected our m6A-immunoprecipitated mRNA and total mRNA fractions to algorithmic m6A peak calling, annotation, and *IDH1^mut^* versus *IDH1^wt^* differential expression using RADAR (44). Volcano plots for raw RADAR output data revealed a higher percentage of significant (*P* < 0.01) m6A transcript sites with an absolute value log_2_ fold change >0.5 in *IDH1^mut^* cells (61%) compared with *IDH1^wt^* (39%; Fig. 1J). To avoid false positives due to cell models, sample size, or m6A hypermethylation lacking functional consequence in patients, we intersected this RADAR m6A list (2,438 genes) to gene found to be differentially expressed (*P* < 1.0 × 10^−6^) with an absolute value log_2_ fold change greater than 1.0 (3,763 genes; Fig. 1K) in TCGA GBM+LGG *IDH^mut^* and *IDH^wt^* RNA-seq patient data. This yielded a list of MeRIP-seq RADAR m6A targets (217 genes) with high clinical relevance. Genes in this list that also conformed to m6A functional consequences (i.e., RADAR m6A was associated with decreased transcript expression in TCGA data) were plotted as a supervised heat map (Fig. 1L). One of the genes from this heatmap was *ATF5*, a critical apoptosis transcription factor with implications in glioma (34, 35), which we explore below. We looked for motifs within the MeRIP-seq fragments and found DRACH m6A consensus sites as expected. In addition, we found that 1/3 had SFPQ binding motif (CUGUG; ref. 57). This unbiased screen of m6A enriched transcripts provides evidence for an increase in m6A-containing transcripts in *IDH1^mut^* glioma cell lines relative to *IDH1^wt^* and suggests the presence of a m6A hypermethylation phenotype in *IDH1^mut^* glioma [which we propose to name as G-RAMP (*IDH1^mut^*glioma RNA m6-adenosine hypermethylation phenotype), similar to G-CIMP].

### *IDH1^mut^* Gliomas Produce D-2-HG That Attenuates Glioma Cell Proliferation and Increases Apoptosis

**FIGURE 2:**
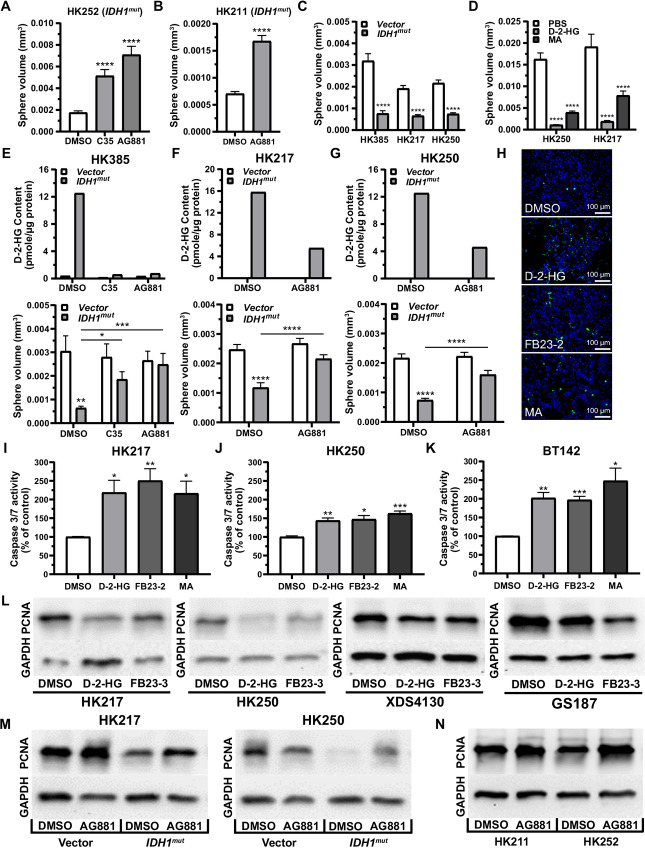
IDH1^mut^ production of D-2-HG inhibits gliomasphere growth. IDH1^mut^ inhibitors (C35 = 2 µmol/L; AG881 = 1 µmol/L) enhance growth of *IDH1^mut^*patient-derived gliomaspheres HK252 (**A**) and HK211 (**B**). **C,** Effect of *IDH1^mut^* forced expression in *IDH1^wt^* gliomaspheres (HK385, HK217, and HK250) on gliomasphere growth relative to vector control [ANOVA, *F*(1,1056) = 199.0, *P* ≤ 0.0001; asterisks indicate *post hoc* Newman–Keuls comparison with vector], showing decreased overall growth in *IDH1^mut^*. **D,** Octyl-D-2-HG (0.5 mmol/L) and FTO inhibitor MA (100 µmol/L) treatment inhibited growth of *IDH1^wt^* gliomaspheres (HK217 and HK250) [ANOVA, *F*(2,996) = 80.4, *P* ≤ 0.0001; asterisks indicate *post hoc* Newman–Keuls comparison of octyl-D-2-HG or MA with PBS treatment]. Top: IDH1^mut^ inhibitor (C35 = 2 µmol/L, or AG881 = 1 µmol/L) treatment reduces intracellular D-2-HG content (*n* = 1) in *IDH1^wt^* gliomaspheres [HK385 (**E**), HK217 (**F**), HK250 (**G**)] with *IDH1^mut^* forced expression. D-2-HG levels in untreated vector gliomaspheres were below detection threshold and were not visible. Bottom: *IDH1^wt^* gliomaspheres with *IDH1^mut^* forced expression exhibited differences in gliomasphere growth [ANOVA, E: *F*(1,353) = 9.6, *P* ≤ 0.002, F: *F*(1,890) = 30.6, *P* ≤ 0.0001, G: *F*(1,833) = 73.0, *P* ≤ 0.0001], and gliomasphere growth. *IDH1^mut^* forced expression spheres could be enhanced with IDH1^mut^ inhibitor treatment [ANOVA, E: *F*(2,353) = 1.3, *P* ≤ 0.3, F: *F*(1,890) = 13.1, *P* ≤ 0.0003, G: *F*(1,833) = 14.8, *P* ≤ 0.0001]. Asterisks indicate *post hoc* Newman–Keuls comparisons between *IDH1^mut^* and vector, or between groups indicated by the horizontal bars. **H,** Treatment with octyl-D-2-HG (1.0 mmol/L) or FTO inhibitors (FB23–2 = 3 µmol/L; MA = 100 µmol/L) appeared to induce apoptosis (green fluorescence), but not necrosis (red fluorescence), in U87 cells using an Abcam Apoptosis/Necrosis Assay Kit (10X magnification). Quantification of increased apoptotic Caspase 3/7 activity (detected with Promega Caspase-Glo 3/7 Assay kit) in *IDH1^wt^* (**I**, **J**) and hemizygous *IDH1^mut/^*^−^ BT142 (**K**) gliomaspheres following treatment with octyl-D-2-HG (0.5 mmol/L) or FTO inhibitor (FB23–2 = 3 µmol/L; MA = 100 µmol/L). **L,** Octyl-D-2-HG (0.5 mmol/L) or FTO inhibitor (FB23–2 = 3 µmol/L) treatment reduced PCNA expression in Western blots of *IDH1^wt^* gliomaspheres. **M,***IDH1^wt^* gliomaspheres (HK217 and HK250) exhibiting *IDH1^mut^* forced expression demonstrated reduced PCNA expression via Western blot analysis. Treatment with IDH1^mut^ inhibitor (AG881 = 1 µmol/L) was able to restore PCNA expression in *IDH1^mut^*gliomaspheres, but not in vector controls. **N,** IDH1^mut^ inhibitor (AG881 = 1 µmol/L) treatment enhanced PCNA expression in native *IDH1^mut^* patient-derived gliomaspheres (HK211, HK252). PCNA protein expression was determined relative to GAPDH protein loading controls. *, *P* ≤ 0.05; **, *P* ≤ 0.01; ***, *P* ≤ 0.001; and ****, *P* ≤ 0.0001 compared with relevant controls. Unless otherwise stated, *P* values indicate unpaired Student *t* test comparisons with the control, or between two groups as indicated by the horizontal line.

To confirm slower tumor growth in *IDH1^mut^* glioma and understand the underlying mechanism, we measured the effects of *IDH1^mut^* and D-2-HG on glioma cell proliferation and quantified related alterations in m6A abundance. Native heterozygous *IDH1^mut^* gliomaspheres (HK252 and HK211) treated with IDH1^mut^ inhibitors demonstrated reductions in intracellular D-2-HG and m6A modifications (Fig. 1H and I), but gliomasphere growth was markedly enhanced as evidenced by increased sphere volumes (Fig. 2A and B). Hemizygous *IDH1^mut/^^−^* BT142 gliomaspheres exhibiting nearly undetectable level of D-2-HG (Supplementary Fig. S1A), demonstrate unchanged m6A expression upon treatment with AG881 or C35 via dot blot (Supplementary Fig. S1I); as expected, we detected no change in sphere growth upon IDH1^mut^ inhibition (Supplementary Fig. S2A). Furthermore, cell growth in native *IDH1^wt^* gliomaspheres (HK385, HK217, and HK250) was rapidly attenuated upon forced *IDH1^mut^* expression (Fig. 2C), and octyl-D-2-HG treatment (0.5 mmol/L) alone acted as a potent inhibitor of growth in native *IDH1^wt^* gliomaspheres (Fig. 2D). Reduced viability was octyl-D-2-HG dose dependent in all tested cell lines, including *IDH1^wt^* gliomaspheres, NHA cells, and U87 cells (Supplementary Fig. S2B–S2E). Growth attenuation effects also proved to be reversible when glioma cells were treated with C35 and AG881 to reduce D-2-HG (Fig. 2E–G, top) causing partial restoration of *IDH1^mut^* gliomasphere proliferation while having no obvious effect on vector control spheres (Fig. 2E–G, bottom). Similarly, in *IDH1^mut^*-expressing U87 cells, we observed that the decreased cell viability compared with control U87 cells could be partially reversed by C35 (Supplementary Fig. S2F).

To determine whether increased apoptosis or necrosis contributed to decreased growth, we also used the Apoptosis/Necrosis Assay kit (Abcam) to differentiate apoptotic cells from those exhibiting slower proliferation rates. As shown in Fig. 2H, U87 cells treated with 1 mmol/L octyl-D-2-HG for 72 hours qualitatively exhibited increased apoptosis compared with DMSO-treated control cells, suggesting that inhibition of FTO by D-2-HG induces apoptosis without having a direct cytotoxic effect as no necrotic cells were detected. To confirm this effect on apoptosis, we employed the Caspase-Glo 3/7 Assay kit (Promega) to quantify changes in apoptotic mediators in gliomasphere cell lines. Native *IDH1^wt^* gliomaspheres (HK217 and HK250) and hemizygous *IDH1^mut/^^−^* BT142 gliomaspheres demonstrated consistent Caspase 3/7 activation following octyl-D-2-HG (0.5 mmol/L) treatment (Fig. 2I–K). Finally, to test whether D-2-HG can alter cell proliferation through non-apoptotic mechanisms, we also examined expression levels of the cellular proliferation marker PCNA (proliferating cell nuclear antigen) via Western blot analysis. Native *IDH1^wt^* gliomaspheres (HK217, HK250, XDS4130, and GS187) treated with 0.5 mmol/L octyl-D-2-HG demonstrated reduced PCNA levels compared with DMSO-treated controls (Fig. 2L). Ectopic expression of *IDH1^mut^* in HK217 and HK250 gliomaspheres induced a similar decrease in PCNA (Fig. 2M). As expected, pharmacologic inhibition of D-2-HG generation by AG881 (1 µmol/L) not only increased PCNA expression in native *IDH1^mut^* gliomaspheres (HK211 and HK252; Fig. 2N), but also restored PCNA expression levels in *IDH1^mut^*-expressing HK217 and HK250 gliomaspheres (Fig. 2M). These results indicate that *IDH1^mut^* expression contributes to slower growing phenotype by decreasing cell proliferation while increasing apoptosis in a subset of cells in a D-2-HG–dependent fashion.

### FTO Inhibition Mediates m6A Hypermethylation and Growth Inhibition in *IDH1^wt^* U87 and Gliomaspheres

**FIGURE 3:**
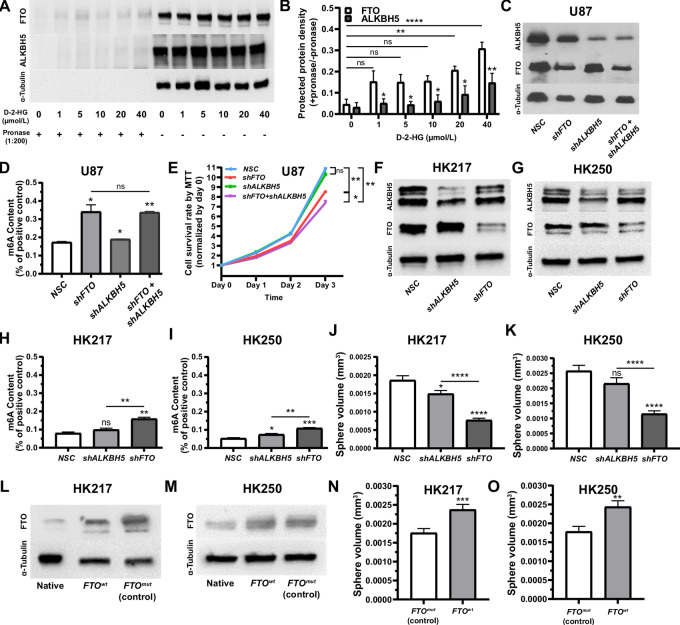
Role of m6A demethylases FTO and ALKBH5 in mediating m6A hypermethylation and growth inhibition. **A,** Western blot images of FTO, ALKBH5 and α-tubulin protein content following DARTS assay in U87 cells. **B,** Normalized densities of Western blots in A, showing increased protected protein density in FTO lysates treated with D-2-HG over ALKBH5. The effect of FTO versus ALKBH5 lysates on protected protein density was evaluated via ANOVA [*F*(1,30) = 27.2, *P* ≤ 0.0001; asterisks indicate *post hoc* Newman–Keuls comparison within treatment of D-2HG, or between groups as indicated by the horizontal bars]. The densities of FTO and ALKBH5 DARTS products were normalized with parallel FTO and ALKBH5 Western blot densities of mock DARTS products (no protease added). **C,** Western blot analysis showing downregulation of FTO and ALKBH5 following shRNA knockdown in U87. **D,** Greater effect of *FTO* than *ALKBH5* knockdown (shRNA) on increasing m6A content in U87. Treatment with *shFTO* and *shALKBH5* was similar to *shFTO* only treatment. **E,** MTT assays demonstrating that shRNA treatment (*shFTO*, or *shFTO and shALKBH5*) reduces the viability of U87 cell lines [ANOVA, *F*(3,8) = 39.1, *P* ≤ 0.0001]. Treatment effect was heterogenous regarding *FTO* and *ALKBH5*, with *shFTO* demonstrating an effect on cell viability and *shALKBH5* having no effect in comparison with a scrambled sequence (NSC; *post hoc* Newman–Keuls comparisons, indicated by the asterisks and brackets). Western blots showing downregulation of FTO and ALKBH5 following shRNA knockdown in HK217 (**F**) and HK250 (**G**) gliomaspheres. *FTO* knockdown (shRNA) has a greater effect on m6A content increases than *ALKBH5* in HK217 (**H**) and HK250 (**I**) gliomaspheres. *FTO* knockdown (shRNA) is more effective at reducing sphere growth than *ALKBH5* in HK217 (**J**) and HK250 (**K**) gliomaspheres. Western blots showing successful overexpression of FTO^wt^ and inactivated FTO^mut^ control in HK217 (**L**) and HK250 (**M**) gliomaspheres. *FTO^wt^* overexpression increases gliomasphere growth in comparison with inactivated FTO^mut^ control in HK217 (**N**, *P* ≤ 0.0006) and HK250 (**O**, *P* ≤ 0.002) gliomaspheres. *, *P* ≤ 0.05; **, *P* ≤ 0.01; ***, *P* ≤ 0.001; and ****, *P* ≤ 0.0001 compared with relevant controls. Unless otherwise stated, *P*-values indicate unpaired Student *t* test comparisons with the control, or between two groups as indicated by the horizontal line.

Treatment with the FTO-specific inhibitor MA (100 µmol/L) resulted in similar reductions in *IDH1^wt^* gliomasphere growth as seen for D-2-HG (Fig. 2D; ref. 14). We found that FTO-specific pharmacologic inhibitors FB23-2 and MA resulted in a similar of both apoptosis induction and proliferation inhibition in *IDH1^wt^* gliomaspheres (Fig. 2H–L), suggesting that FTO may be a critical target of D-2-HG whose inhibition can reduce tumor growth (14, 25). We tested the relative affinity of D-2-HG for FTO versus ALKBH5 using DARTS assays (42). Western blot analysis (Fig. 3A) and its quantification (Fig. 3B) demonstrate that D-2-HG interacts with FTO and ALKBH5 in a dose-dependent manner. Importantly, the D-2-HG concentration necessary to achieve protein stability in the setting of proteases is at least 10 times higher for ALKBH5 versus FTO, suggesting D-2-HG exhibits a relative selective binding affinity for FTO. This supports the conclusion that D-2HG directly inhibits FTO demethylase activity as previously shown (26).

We performed selective short hairpin RNA (shRNA) knockdown experiments to further assess the possibility that FTO, rather than ALKBH5, is the main mediator of D-2-HG effects on m6A in glioma. U87 cells were transfected with lentivirus harboring piLenti-*FTO-shRNA*, -*ALKBH5-shRNA*, -*FTO-shRNA+ALKBH5-shRNA*, or a piLenti-*NSC-shRNA* scramble control (NSC). An integrated GFP reporter was used to confirm successful transfection (Supplementary Fig. S3A–S3C). Interestingly, despite successful FTO and ALKBH5 knockdown (Fig. 3C), only *FTO* shRNA resulted in the expected phenotypes of increased m6A abundance and reduced cell viability. *ALKBH5* shRNA had no appreciable effect in this regard, and concurrent knockdown of both *FTO* and *ALKBH5* merely recapitulated the effects seen with *FTO* shRNA (Fig. 3D and E). Similarly, upon introduction of *FTO*- and *ALKBH5*-targeting shRNA in *IDH1^wt^* gliomaspheres (HK217 and HK250) and confirmation of reduced FTO and ALKBH5 protein expression (Fig. 3F and G), we found that *ALKBH5* knockdown had less effect than *FTO* knockdown in increasing in m6A abundance (Fig. 3H and I). Accordingly, *FTO* knockdown more effectively inhibited gliomasphere growth (Fig. 3J and K). We also performed FTO and ALKBH5 overexpression experiments using lentiviral vectors carrying pMIRNA1-*FTO^wt^-GFP* or -*FTO^mut^-GFP*, as well as pCDH-*ALKBH5^wt^-GFP* or -*ALKBH5^mut^-GFP*. *FTO^mut^* and *ALKBH5^mut^* encoding enzymatically inactive FTO and ALKBH5 proteins were employed as controls (15). Western blots confirmed overexpression of FTO^wt^ and FTO^mut^ in both HK217 and HK250 (Fig. 3L and M). FTO^wt^ overexpression led to enhanced gliomasphere growth rates in native *IDH1^wt^* gliomaspheres (HK217, HK250) compared with FTO^mut^ (Fig. 3N and O). In contrast, FTO^wt^ overexpression did not lead to enhanced proliferation compared with FTO^mut^ in a native *IDH1^mut^* gliomaspheres line (HK252; Supplementary Fig. S3D), likely due to the presence of baseline inhibition of FTO enzymatic activity by D-2-HG. In contrast to the FTO overexpression experiments in *IDH1^wt^* gliomaspheres, the same gliomaspheres did not show increased growth of ALKBH5^wt^ versus ALKBH5^mut^ overexpression (Supplementary Fig. S3E and S3F). The knockdown and overexpression results indicate that FTO may play a more important role in regulating glioma cell proliferation. Coupled with the higher affinity of D-2-HG for FTO versus ALKBH5 at the molecular level, our experiments indicate that IDH1^mut^-generated D-2-HG inhibits FTO to induce m6A hypermethylation that ultimately results in the slower growth phenotype seen in *IDH1^mut^* gliomas.

### Pharmacologic Inhibition of FTO Reduces Glioma Cell Growth in *IDH1^wt^* Glioma Cells *In Vitro* and *In Vivo*

**FIGURE 4:**
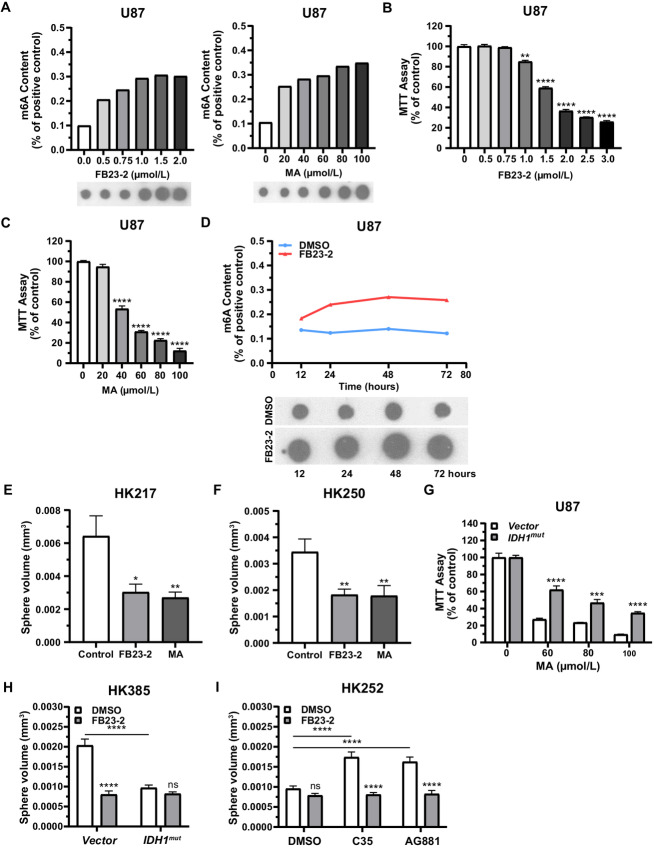
FTO inhibition attenuates gliomasphere growth. **A,** Increase in U87 RNA m6A content relative to control (0 µmol/L) following the application of FTO inhibitors FB23-2 (left) or MA (right) at various doses. Dose-dependent inhibition of U87 cell viability by FTO inhibitors FB23-2 (**B**) and MA (**C**) evaluated by MTT assay. **D,** Time course of m6A enrichment in U87 cells treated with the FTO inhibitor FB23-2 (3 µmol/L, red) or DMSO (blue). One representative dot blot comparison between FB23-2–treated or DMSO-treated U87 cells is shown below the graph. Growth inhibition following FTO inhibitor treatment (FB23-2 = 3 µmol/L; MA = 100 µmol/L) in *IDH1^wt^* HK217 (**E**) and HK250 (**F**) gliomaspheres. **G,** Treatment of both vector control and *IDH1^mut^* forced expressing U87 cells with MA results in decreased viability (ANOVA, *F*(3,16) = 271.4, *P* ≤ 0.0001; asterisks indicate *post hoc* Newman–Keuls comparisons at each dose between *IDH1^mut^* and vector). **H,** Treatment of HK385 gliomaspheres with the FTO inhibitor FB23-2 (3 µmol/L) attenuates sphere growth in vector gliomaspheres but not those exhibiting *IDH1^mut^* forced expression [ANOVA, treatment: *F*(1,937) = 51.1, *P* ≤ 0.0001; vector vs. *IDH1^mut^*: *F*(1,937) = 29.4, *P* ≤ 0.0001; interaction: *F*(1,937) = 31.3, *P* ≤ 0.0001; asterisks indicate *post hoc* Newman–Keuls comparison with DMSO or between groups as indicated by horizontal bars]. **I,** IDH1^mut^ inhibitor (C35 = 2 µmol/L; or AG881 = 1 µmol/L) treatment enhanced growth in *IDH1^mut^* forced expression HK252 gliomaspheres, thereby restoring sensitivity to FB23-2 (3 µmol/L) [ANOVA, IDH^mut^ inhibitor treatment: *F*(2,1133) = 10.9, *P* ≤ 0.0001; FTO inhibitor treatment: *F*(1,1133) = 73.7, *P* ≤ 0.0001; interaction: *F*(2,1133) = 9.5, *P* ≤ 0.0001; asterisks indicate *post hoc* Newman–Keuls comparison with DMSO or between groups as indicated by the horizontal bars]. *, *P* ≤ 0.05; **, *P* ≤ 0.01; ***, *P* ≤ 0.001; and ****, P ≤ 0.0001 compared with relevant controls. Unless otherwise stated, *P* values indicate unpaired Student *t* test comparisons with the control or between two groups as indicated by the horizontal line.

Having established the predominant role of FTO inhibition versus ALKBH5 inhibition in m6A hypermethylation in *IDH1^wt^* glioma cells, we employed FTO-specific small-molecule inhibitors, FB23-2 and MA, to test whether pharmacologic FTO inhibition might provide a tractable treatment strategy. In U87 cells, FB23-2 and MA induced m6A hypermethylation (Fig. 4A). Western blot analysis confirmed no change in writer or eraser expression with FB23-2 treatment in HK250 cells (Supplementary Fig. S1J). We observed decreased cell viability in a dose-dependent manner achieved with FB23-2 at lower concentrations (1 µmol/L) compared with MA (40 µmol/L; Fig. 4B and C). The increased m6A induced by FB23-2 persisted at least 72 hours posttreatment (Fig. 4D). In *IDH1^wt^* gliomaspheres (HK217 and HK250), sphere growth was reduced following treatment with FB23-2 (3 µmol/L, refreshed every 72 hours) or MA (100 µmol/L, refreshed every 72 hours; Fig. 4E and F). All other tested *IDH1^wt^* gliomaspheres, including GS025, GS062, GS158, GS187, XDS1152, and XDS4130 manifested similar growth inhibition by FB23-2 (Supplementary Fig. S4A–S4F). Native *IDH1^wt^* U87 cells expressing *IDH1^mut^* or vector (control) were treated with pharmacologic doses of MA (Fig. 4G) previously demonstrated to produce m6A hypermethylation (Fig. 4A) and decrease cell viability (Fig. 4C). Vector (control) U87 were more sensitive than those expressing *IDH1^mut^*, although there appeared to be nonspecific toxicity from MA (Fig. 4G). Native *IDH1^wt^* HK385 gliomaspheres expressing *IDH1^mut^* or vector (control) were treated with FB23-2 (Fig. 4H). Again, the *IDH1^wt^* HK385 gliomaspheres showed decreased cell growth following FB23-2 treatment, in contrast to *IDH1^mut^* HK385 gliomaspheres, which showed unchanged cell growth.

Similarly, growth inhibition was not seen in native *IDH1^mut^* HK252 gliomaspheres treated with FB23-2, as might be expected in the context of baseline FTO inhibition by endogenous D-2-HG causing “resistance” (Fig. 4I). However, increased growth induced by the IDH1^mut^ inhibitors AG881 and C35 was neutralized by FB23-2 cotreatment (Fig. 4I). As expected, hemizygous *IDH1^mut/^^−^* BT142 spheres (with low baseline D-2-HG) and *IDH1^wt^* control HK385 gliomaspheres were sensitive to FB23-2 growth inhibition and remained unaffected by cotreatment with IDH1^mut^ inhibitors (Supplementary Fig. S4G and S4H). These results indicate that the FTO inhibitor may be more effective in the absence of D-2-HG, which is found in *IDH1^wt^* gliomas.

**FIGURE 5:**
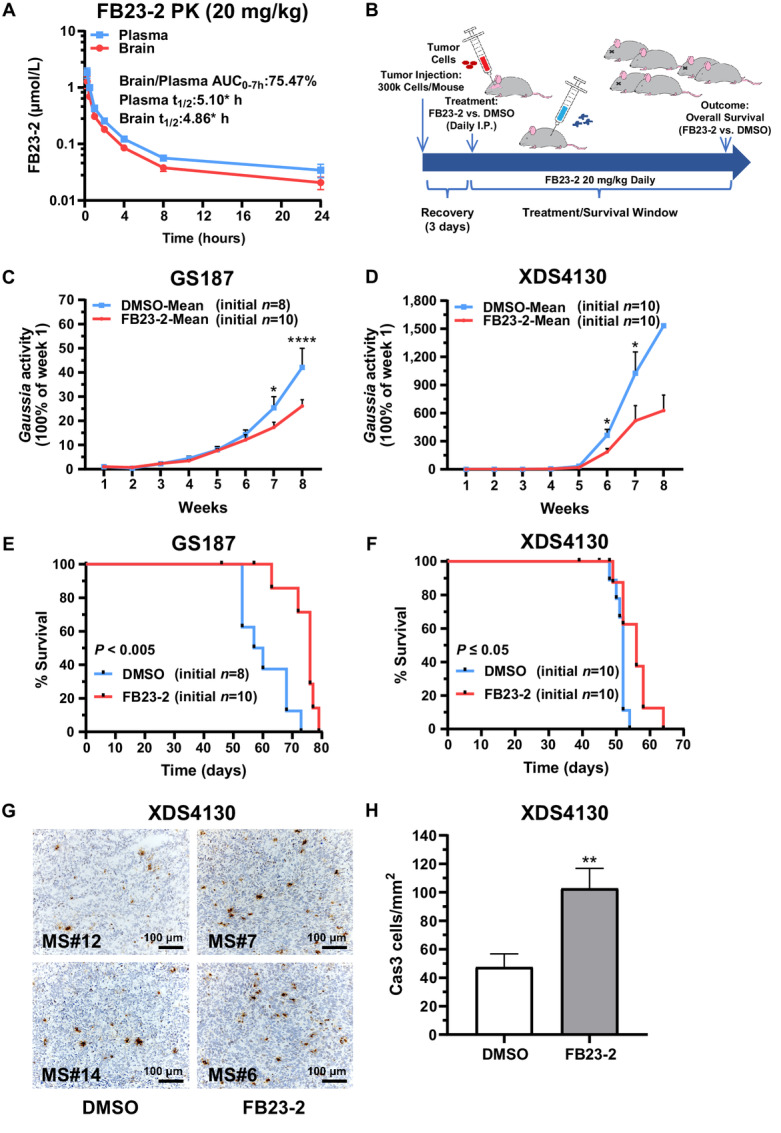
Pharmacologic inhibition of FTO using FB23-2 reduces tumor growth rates in intracranial *IDH1^wt^* gliomasphere xenografts. **A,** Pharmacokinetic analysis of FB23-2 permeability through the BBB showing drug elimination dynamics in plasma and brain (2 mice/timepoint). **B,** Schematic of *in vivo* experiments whereby intracranial gliomasphere xenografts were established before undergoing randomization to daily intraperitoneal injections of either FB23-2 treatment or DMSO control, with injection volumes based on individual weights (20 mg/kg). *In vivo* monitoring of tumor growth measured via plasma *Gaussia* Luciferase activity (tail vein) revealed differences at 7–8 weeks (GS187) and 6–7 weeks (XDS4130; at week 8, only 1 mouse remained in DMSO group), respectively [ANOVA, (**C**) treatment: *F*(1,16) = 4.7, *P* ≤ 0.05; time: *F*(7,106) = 79.1, *P* ≤ 0.0001; interaction: *F*(7,106) = 4.3, *P* ≤ 0.0003; (**D**) treatment: *F*(1,18) = 16.7, *P* ≤ 0.0007; time: *F*(1.6,23.6) = 41.7, *P* ≤ 0.0001; interaction: *F*(7,104) = 5.3, *P* ≤ 0.0001; asterisks indicate *post hoc* Newman–Keuls on day 7 and 8]. Kaplan–Meier curves showing overall survival differences in xenograft mice treated with FB23-2 (3 µmol/L) or DMSO control, evaluated via a log-rank (Mantel–Cox) test for GS187 (**E**, *P* < 0.005) and XDS4130 (**F**, *P* ≤ 0.05). **G,** Activated Cas3 immunostaining (**G**, 10X magnification) of representative XDS4130 xenografts (surgically extracted and flash-frozen post-mortem). “MS” refers to the mouse identification number. **H,** Increase in number of Cas3 positive cells in tumors from mice treated with FB23-2 relative to DMSO controls (*P* ≤ 0.005). *, *P* ≤ 0.05; **, *P* ≤ 0.01; ***, *P* ≤ 0.001; and ****, *P* ≤ 0.0001 compared with relevant controls. Unless otherwise stated, *P* values indicate unpaired Student *t* test comparisons with the control, or between two groups as indicated by the horizontal line.

To investigate this therapeutic avenue *in vivo*, we tested the permeability of FB23-2 across the blood–brain barrier (BBB) in mice (2 mice/timepoint). Intraperitoneal injection of FB23-2 at a dose of 20 mg/kg, determined to have no harmful side effects in mice (25), achieved FB23-2 concentrations in plasma and brain tissue above 1 µmol/L, with an estimated 75.5% uptake across the BBB based on AUC_0–7hr_ (Fig. 5A). This dose of FB23-2 (or an equivalent volume of DMSO) was administrated daily to mice beginning on day 4 following intracranial xenografting of *IDH1^wt^* gliomaspheres (GS187 and XDS4130), with the subsequent treatment course lasting for 60 days (Fig. 5B). These gliomaspheres had been labeled with GLuc, a convenient reporter for noninvasively monitoring *in vivo* tumor growth using simple blood draws (58). As shown in Fig. 5C and D, GLuc activity in blood samples (two tail vein collections per mouse per week) normalized internally to the first sample collected 7 days following intracranial xenografting increased steadily over time. The rate of GLuc increase was slower in FB23-2–treated mice, achieving separation beginning at week 6 in both GS187 and XDS4130 intracranially xenografted mice. FB23-2 treatment also led to increased overall survival in both GS187 and XDS4130 mice (Fig. 5E and F). log-rank (Mantel–Cox) tests were used to compare survival curves between FB23-2 and DMSO treatment groups. In GS187 xenografted mice treated with FB23-2 (*n* = 10) or DMSO (*n* = 8), log-rank analysis showed a significant difference in overall survival (*P* ≤ 0.001; df = 1; *χ*^2^ = 10.67). Median survival for GS187 mice treated with FB23-2 was 76 days and 58 days for DMSO-treated mice [ratio = 1.3, 95% confidence interval (CI) = 0.47–3.58; HR 0.26, 95% CI = 0.077–0.86]. XDS4130 xenografted mice treated with FB23–2 (*n* = 10) or DMSO (*n* = 10) also showed minor differences in overall survival following log-rank analysis (*P* = 0.01; df = 1; *χ*^2^ = 6.35). Median survival for XDS4130 mice treated with FB23-2 was 56 and 52 days for DMSO-treated mice (ratio = 1.1, 95% CI = 0.42–2.79; HR = 0.42, 95% CI = 0.15–1.16).

Activated Caspase-3 (Cas3) immunostaining of XDS4130 xenografts (flash-frozen post-mortem) showed increased numbers of Cas3-positive cells in tumors treated with FB23-2 (Fig. 5G and H). This indicates increased apoptotic activity in FB23-2–treated gliomas *in vivo*, a finding consistent with our *in vitro* experiments (Fig. 2H–K). Notably, FB23-2 treatment did not appear to induce apoptosis of non-tumor cells in post-mortem immunohistologic assessments. Taken together, these experiments show that pharmacologic inhibition of FTO reliably reduces *IDH1^wt^* glioma cell growth rates both *in vitro* and *in vivo*.

### The *IDH1^mut^* → D-2-HG ⊣ FTO Axis Leads to *ATF5* mRNA m6A Hypermethylation and Decreased Expression

**FIGURE 6:**
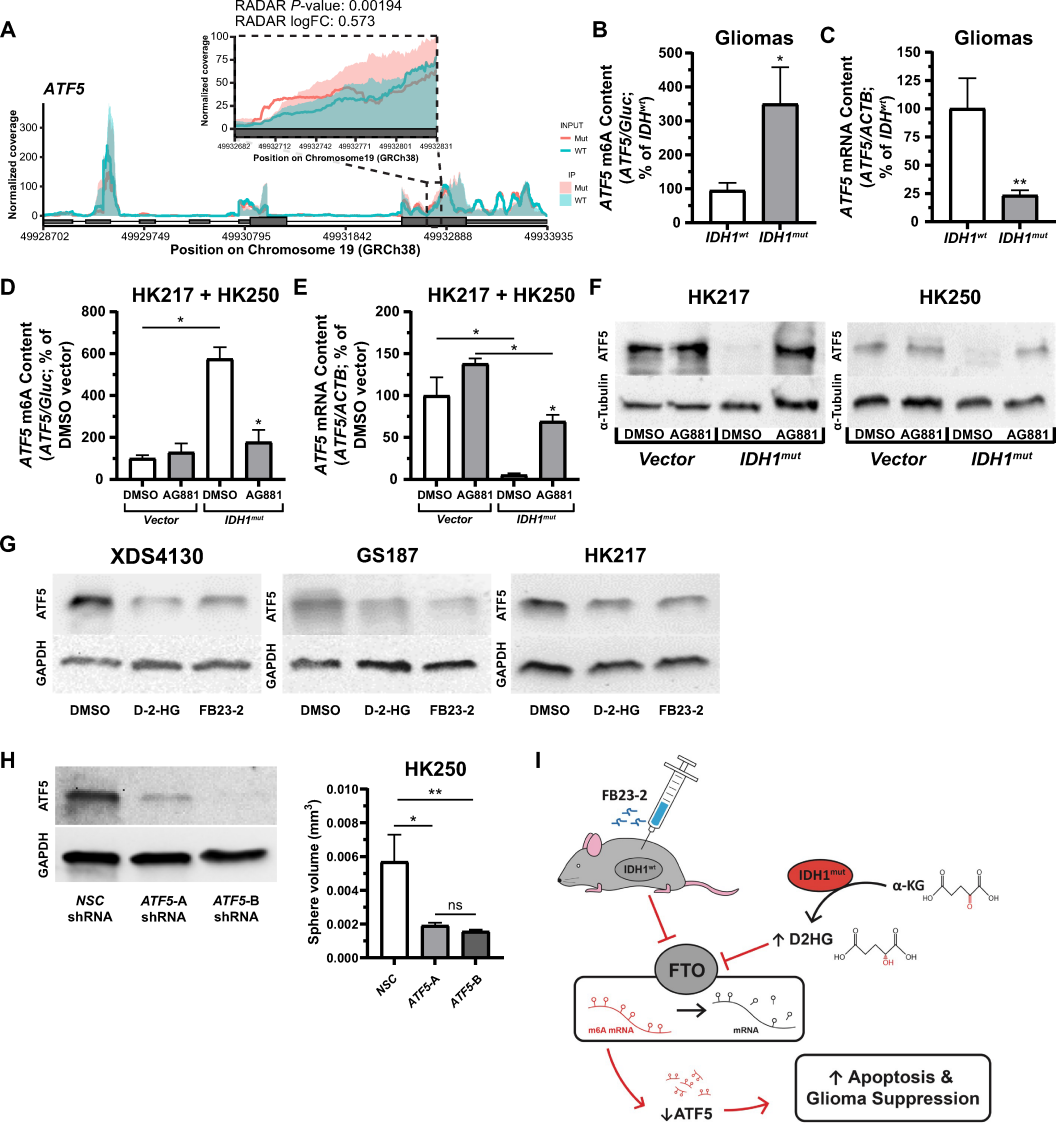
ATF5 is a downstream mediator of the *IDH1^mut^* → D-2-HG ⊣ FTO axis leading to gliomasphere growth reduction. **A,** m6A called peaks of *ATF5* mRNA from MeRIP-seq RADAR data aligned to the genome (GRCh38). Inset graph shows m6A enrichment in *IDH1^mut^* (salmon) versus *IDH1^wt^* (teal) glioma samples for a select coding (exon) region, indicated by the dashed box. IP indicates immunoprecipitated mRNA fraction containing m6A-enriched RNA. INPUT mRNA fraction indicates total mRNA. **B,** Increased m6A-enriched *ATF5* mRNA content in patient *IDH1^mut^* gliomas (*n* = 13) detected by m6A immunoprecipitation qRT-PCR, compared with *IDH1^wt^* gliomas (*n* = 10) (*P* ≤ 0.05). **C,** Decreased *ATF5* mRNA m6A content detected by *ATF5* qRT-PCR in the same *IDH1^mut^* gliomas (*P* ≤ 0.008). **D,** Pooled data from *IDH^wt^* gliomaspheres (HK217 and HK250) showing induction of *ATF5* mRNA m6A enrichment following *IDH1^mut^* forced expression and its subsequent attenuation following treatment with IDH1^mut^ inhibitor AG881 (1 µmol/L). **E,** Pooled data from *IDH^wt^* gliomaspheres (HK217 and HK250) showing a reduction of *ATF5* mRNA expression following *IDH1^mut^* forced expression and *ATF5* mRNA expression rescue following AG881 (1 µmol/L) treatment. **F,** ATF5 protein levels are decreased in gliomaspheres following *IDH1^mut^* forced expression and are rescued following treatment with IDH1^mut^ inhibitor AG881 (1 µmol/L). ATF5 protein expression was determined relative to α-tubulin protein loading controls. **G,** Octyl-D-2-HG (0.5 mmol/L) or FTO inhibitor FB23-2 (3 µmol/L) treatment reduced ATF5 protein expression in *IDH1^wt^* gliomaspheres. **H,***IDH1^wt^* gliomaspheres (HK250) transfected with piLenti-*siRNA-GFP*, expressing shRNA for either NSC or two regions (A, B) of *ATF5*, show reduced ATF5 expression via Western blot analysis (left) and reduced sphere growth (right). **I,** Schematic representation of the *IDH1^mut^* ⊣ FTO axis resulting in m6A enrichment and subsequent degradation of *ATF5* mRNA transcripts leading to increased apoptosis. (*P* ≤ 0.005). *, *P* ≤ 0.05; **, *P* ≤ 0.01; ***, *P* ≤ 0.001; and ****, *P* ≤ 0.0001 compared with relevant controls. Unless otherwise stated, *P* values indicate unpaired Student *t* test comparisons with the control, or between two groups as indicated by the horizontal line.

To understand the relatively slower growth of *IDH1^mut^* gliomas, we hypothesized that specific m6A hypermethylated candidate targets could lead to downregulation of growth pathways. To evaluate this, we conducted MeRIP analysis of differential m6A expression in mRNA between *IDH^mut^* and *IDH^wt^* cell lines and gliomaspheres using RADAR, intersecting these results with TCGA RNA-seq patient data (see Materials and Methods). *ATF5* (activating transcription factor 5) emerged as a promising target for downstream study based on our MeRIP profiling cut-off criteria (Fig. 6A). ATF5 belongs to the CREB (cAMP response element-binding) family of proteins, a subset of the bZIP (basic zipper) protein family, and is primarily involved in cell proliferation, apoptosis, neural stem cell differentiation, and cell responses to stress (34, 35). *ATF5* is also highly expressed in a variety of cancer types, such as glioma, breast cancer, lung cancer, and others. In addition, GlioVis analysis of TCGA RNA-seq data showed that *ATF5* expression was decreased over 2-fold in the *IDH1^mut^* (*n* = 429) glioma patient cohort when compared with the *IDH1^wt^* (*n* = 233) cohort in a pairwise *t* test (*P* < 6.6E-65, with Bonferroni multiple testing correction; Supplementary Fig. S5A). Median *ATF5* mRNA expression for the cohorts was log_2_ 8.36 in *IDH1^mut^* gliomas (SD = 0.66), and log_2_ 9.54 in *IDH1^wt^* gliomas (SD = 0.80), and lower *ATF5* expression was correlated with survival benefits in both *IDH1^mut^* and *IDH1^wt^* glioblastomas (log rank *P* < 0.05; Supplementary Fig. S5B).

To confirm the differences in *ATF5* expression observed in TCGA data and our MeRIP-seq hypermethylation data, we performed targeted qRT-PCR on RNA isolated from fresh frozen glioma tumor samples obtained through the UCLA Brain Tumor Translational Resource. Total RNA was purified from *IDH1^mut^* (*n* = 12) and *IDH1^wt^* (*n* = 12) patient tumor samples and subjected to direct *ATF5* qRT-PCR and m6A-RNA immunoprecipitated *ATF5* qRT-PCR. The levels of *ATF5* mRNA and m6A-enriched *ATF5* transcripts were normalized respectively to β-actin (ACTB) transcript levels or GLuc m6A-positive control RNA that was spiked into samples prior to immunoprecipitation. The levels of m6A-enriched *ATF5* transcripts and total *ATF5* mRNA were determined via qRT-PCR. Prior to stratifying results based on *IDH1* status, the levels of m6A-enriched *ATF5* transcripts were first normalized to a known concentration of GLuc m6A-positive control RNA that was spiked into samples prior to immunoprecipitation. Total *ATF5* mRNA levels were normalized to β-actin (ACTB) transcript levels. In m6A-immunoprecipitated RNA fractions, there was significantly more m6A enrichment in *ATF5* transcripts isolated from *IDH1^mut^* versus *IDH1^wt^* patient tumor samples (*P* ≤ 0.05; Fig. 6B). Increased m6A enrichment was associated with significantly decreased overall abundance of *ATF5* mRNA in *IDH1^mut^*glioma samples (*P* ≤ 0.008; Fig. 6C). Indeed, when *IDH1^mut^* was expressed in native *IDH1^wt^* gliomaspheres (HK217 and HK250), m6A-enriched *ATF5* transcripts increased while overall *ATF5* transcripts decreased (Fig. 6D and E). This change was reversible following treatment with IDH1^mut^ inhibitor, AG881 (1 µmol/L). We confirmed these results using the antibody-independent assay, MazF-qRT-PCR (47), showing increased methylation at specific m6A sites in *IDH1^mut^* patient tissue samples (Supplementary Fig. S5C) and *IDH1^wt^* gliomaspheres with *FTO* shRNA knockdown (Supplementary Fig. S5D) or treated with octyl-D-2-HG or FB23-2 (Supplementary Fig. S5E).

Finally, we confirmed that ATF5 protein expression by Western blot analysis was congruent with transcript changes. We confirmed that the generated *IDH1^mut^* gliomaspheres (HK217, HK250) showed reduced protein expression compared with vector control that could be restored by treatment with AG881 (Fig. 6F). Octyl-D-2-HG (0.5 mmol/L) and FB23-2 (3 µmol/L) treatments in *IDH1^wt^* gliomaspheres were found to reduce ATF5 protein levels (XDS4130, GS187, and HK217; Fig. 6G). Finally, we found that *ATF5* knockdown can attenuate sphere growth in the *IDH1^wt^* gliomasphere line, HK250 (Fig. 6H).

Considered together, our findings in patient-derived gliomaspheres provide evidence in support of our hypothesis that *IDH1^mut^* → D-2-HG ⊣ FTO axis leads directly to m6A hypermethylation of *ATF5* mRNA. Because of ATF5’s role as a critical transcription factor involved in cellular growth and apoptosis (34, 35), this may explain, in part, the slower growth phenotype *of IDH1^mut^* gliomas.

## Discussion

Although *IDH* mutation appear to provide an oncogenic trigger of gliomagenesis, clinical data have shown a strong association between *IDH1* mutations and less aggressive glioma phenotypes exhibiting slower tumor growth (4–7). This study confirms that D-2-HG suppresses glioma growth in a variety of model systems and demonstrates that a contributing mechanism involves D-2-HG–mediated inhibition of the RNA m6A demethylase FTO. This results in m6A enrichment in transcripts such as *ATF5*, whose downregulation is associated with increased apoptosis and/or growth suppression. On the basis of this, we provide evidence for the use of FB23-2, a specific FTO inhibitor, in the treatment of *IDH^wt^* gliomaspheres without inhibition of other α-KG–dependent dioxygenases. Overall, these findings allow us to propose a model diagrammed in Fig. 6I.

We utilized patient tumor samples, patient-derived gliomaspheres, and intracranial xenografts to measure the effects of *IDH1^mut^* on m6A levels and tumor growth. Our study demonstrated that RNA m6A hypermethylation in *IDH1^mut^* gliomas is dependent on D-2-HG generation. We also showed that D-2-HG exposure alone is sufficient to induce m6A hypermethylation in *IDH1^wt^* glioma. Direct inhibition of RNA demethylases using pharmacologic and genetic methods was used to isolate m6A regulatory pathways impacted by *IDH1^mut^* and were then applied to *IDH1^wt^* glioma cells *in vitro* and *in vivo* to observe their effects on tumor growth or viability. We found that altering D-2-HG levels rapidly changes RNA m6A methylation levels, in contrast to D-2-HG–mediated DNA CpG hypermethylation which occurs over longer time courses and is not reversed by the inhibition of D-2-HG generation (59). Our results suggest that D-2-HG dynamically regulates m6A epitranscriptomic alterations and can impact the overall transcriptome and subsequent cellular functions. We also demonstrate that D-2-HG–mediated inhibition of FTO (rather than ALKBH5) is the primary driver of m6A hypermethylation based on: (i) DARTS assays which revealed that FTO exhibits higher affinity for D-2-HG and greater sensitivity to D-2-HG–mediated inhibition than ALKBH5, and (ii) shRNA knockdown experiments showed greater increases in m6A following FTO targeting. The exact mechanisms underlying the dominant influence of FTO in regulating glioma m6A dynamics and cell proliferation remains unclear, but might be explained by differences in catalytic activity, RNA substrate preferences, and cellular localization (60). For example, the catalytic activity of FTO is almost eight times higher (kcat/Km = 0.77 minute^−1^ µmol/L^−1^) than ALKBH5 (kcat/Km = 0.098 minute^−1^ µmol^−1^) in demethylating kinetic analyses performed *in vitro* (61). In addition, FTO is found in both the nucleus and cytoplasm, while ALKBH5 is predominantly found in the nucleus and lacks nuclear export signals (60).

In exploring downstream effectors of our proposed *IDH1^mut^* → D-2-HG ⊣ FTO axis using bioinformatic and biologic experiments, we identified ATF5 (an oncogenic protein involved in proliferation and suppression of apoptosis) as a mediator of glioma growth inhibition. Similar to our data, *ATF5* expression has been shown to be inversely correlated with patient overall survival in glioma (62). Interestingly, a dominant-negative ATF5 isoform has also been shown to induce apoptosis in glioma cells, while having no effect on non-neoplastic neuronal and glial cells (35, 63). The intriguing evidence that *ATF5* exerts prosurvival effects in glioma and not in healthy brain tissue led us to consider whether *ATF5* downregulation, mediated by m6A enrichment of *ATF5* transcripts, might serve as a downstream effector of reduced tumor growth. We discovered that FTO inhibition leads directly to m6A hypermethylation of *ATF5* mRNA, and consequently to reduction in transcript level and protein expression, suggesting a role of ATF5 as a mediator between FTO inhibition and increased apoptosis and reduced tumor cell proliferation in *IDH1^mut^* glioma cells.

To exploit the growth-attenuating effects of FTO inhibition, we tested the possibility of targeting FTO and m6A regulation as a novel treatment strategy for *IDH1^wt^* gliomas. We found that FTO inhibition produced consistent growth suppression *in vitro and in vivo*. In theory, targeting FTO specifically may be preferable to using D-2-HG or other nonspecific α-KG–dependent dioxygenase inhibitors that have undesired pleiotropic effects. The therapeutic implications of our study for the treatment of *IDH1^mut^* gliomas appear more complex, primarily due to the apparent multiplicity of downstream effects of IDH1^mut^ generated D-2-HG. On one hand, IDH1^mut^ generation of D-2-HG can trigger gliomagenesis by interfering with DNA/histone methylation, HIF1α, and DNA damage repair, while on the other acting as a growth suppressor of established gliomas (4–7), via mechanisms such as FTO inhibition.

The “double-edged sword” of D-2-HG may therefore raise the important question about the ultimate role and timing of agents that inhibit IDH1^mut^ production of D-2-HG, available off-label or in clinical trials reviewed elsewhere (64). Although a randomized trial of AG881 versus placebo showed early clinical benefit in the treatment of treatment-naïve, nonenhancing *IDH1^mut^* gliomas (54), our data raise the possibility that D-2-HG reduction alone could have deleterious effects suggesting a role for combination treatment of IDH1^mut^ inhibitor, AG881, and FTO inhibitor. In addition, a subset of *IDH1^mut^* gliomas that recur and subsequently demonstrate rapid growth phenotypes have been shown to produce far lower levels of D-2-HG even while retaining *IDH1* mutations (65). One such mechanism involves a LOH at the *IDH1* locus in which the *IDH1^mut^* allele exists without a wildtype allele following mutant allele amplification. This subset of progressed gliomas exhibiting a loss of *IDH1* heterozygosity (resulting in reduced D-2-HG generation like that seen in BT142 cells) are thus likely to show sensitivity to FTO inhibitors, similarly to *IDH1^wt^* gliomas.

Our experiments do not account for the tumor microenvironment, defining an important area for further study because D-2-HG secreted from *IDH1^mut^* glioma cells may act upon nearby cells, including those mediating immune responses. While our data indicate that D-2-HG is sufficient to mediate growth suppression of glioma cells via m6A pathways, we cannot rule out the possibility that altered tricarboxylic acid cycle metabolism, NADH and α-KG levels may also have a role in *IDH^mut^*-mediated growth suppression (66–68). We also have not included mechanistic investigations of how m6A modifications affect *ATF5* transcript fate and degradation at the transcript level. Our data suggest the presence of a glioma RNA m6A hypermethylation phenotype (G-RAMP) *IDH1^mut^* glioma, analogous to the DNA hypermethylated G-CIMP phenotype that has been described previously (59). However, despite our access to patient glioma samples, definitively establishing G-RAMP in patient samples is currently hindered by the high concentrations of RNA input material required for comprehensive m6A profiling. Another limitation seen in our *in vivo* studies was the ability to translate the robust growth suppression effects seen *in vitro* following treatment with small molecule FTO inhibitors (FB23-2). Studies to engineer FTO-specific small-molecule inhibitors that are better equipped to cross the BBB and maintain robust pharmacokinetic stability are needed. Because our *in vivo* data show only a modest improvement in survival with addition of FB23-2 started at 3 days after implantation, combination of FB23-2 with radiation or chemotherapy may enhance this benefit. Finally, while comparisons between *IDH1^wt^* and *IDH1^mut^* patient tumors and gliomaspheres with disparate genetic backgrounds have been used to establish differences in m6A levels, our mechanistic experiments in which we determine effects of *IDH1* mutation expression or treatment with D-2-HG or IDH inhibitor on glioma cells provide some control for genetic background.

Overall, our study demonstrates how the *IDH1^mut^* → D-2-HG ⊣ FTO axis alters the m6A epitranscriptomic landscape in glioma, affecting downstream factors involved in apoptotic pathways and cellular proliferation suggesting a proposed model that includes *ATF5* (Fig. 6I; refs. 35, 69). We show that this axis can be targeted in aggressive *IDH1^wt^*glioma tumor models and may represent a novel avenue for further therapeutic development.

## Supplementary Material

Supplementary Methods and Materials Supplementary Methods and Materials for detailed information on Immunoblotting, Intracellular D-2-HG Quantification, Gene-specific m6A RT-qPCR, TCGA Gene Expression Analyses, Gliomasphere Microarray Analyses, Animal Procedures, Gaussia Luciferase Tumor Burden Estimation, and Survival Analysis.

Table S1 Glioma Patient Sample Characteristics

Table S2 Patient Derived Glioma Stem Cell Characteristics

Figure S1 IDH1mut Production of D-2-HG Induces RNA m6A Hypermethylation.

Figure S2 Effects of Octyl-D-2-HG and IDH1mut Inhibitors on Glioma Cell Growth.

Figure S3 Effects of FTO and ALKBH5 shRNA Knockdown or Overexpression on Glioma Cell Growth.

Figure S4 Pharmacologic Inhibition of FTO Results in Growth Inhibition of IDH1wt Gliomaspheres In Vitro.

Figure S5 ATF5 is a Downstream Effector of the IDH1mut → D-2-HG ⊣ FTO Axis Resulting in Gliomasphere Growth Reduction.
