# Supplementary material for: D-2-HG Inhibits IDH1mut Glioma Growth via FTO Inhibition and Resultant m6A Hypermethylation
Source: Cancer Res Commun. 2024 Mar 22;4(3):876–94. doi: 10.1158/2767-9764.CRC-23-0271 (PMC10959073; doi:10.1158/2767-9764.CRC-23-0271)
Supplement: Supplementary Methods and Materials — for detailed information on Immunoblotting, Intracellular D-2-HG Quantification, Gene-specific m6A RT-qPCR, TCGA Gene Expression Analyses, Gliomasphere Microarray Analyses, Animal Procedures, Gaussia Luciferase Tumor Burden Estimation, and Survival Analysis. [file crc-23-0271-s01.docx]

**SUPPLEMENTARY METHODS & MATERIALS**

*Immunoblotting*

Cells or tissues were lysed in 1x Pierce RIPA lysis buffer (Thermo Fisher Scientific Cat# 89900) with proteinase inhibitor in a 1:100 dilution (Thermo Fisher Scientific Cat# EO0491). Purified protein content from the lysates was measured using the Pierce BCA Protein Assay Kit (Thermo Fisher Scientific Cat# 23225). Protein was denatured at 100 °C for 10 min with Laemmli Sample Buffer. Equal concentrations of protein were then electrophoretically fractionated in 4–15% precast polyacrylamide gels (8.6 x 6.7 cm, for use with Mini-PROTEAN Electrophoresis Cells) (Bio-Rad Cat# 4568084) and transferred to nitrocellulose membranes. The membranes were then subjected to immunoblot assays following a similar protocol as described above for m6A dot blots. One alteration was that blocking buffer was performed in TBST with 5% dry milk. Specific primary antibodies include rabbit anti-IDH1^mut^ (1:1000) (Dianova Cat# DIA-H09, RRID:AB_2335716), polyclonal rabbit anti-FTO (1:5000) (Novus Cat# NB110-60935, RRID:AB_925405), rabbit anti-ALKBH5 (1:2000) (Novus Cat# NBP1-82188, RRID:AB_11037354) rabbit anti-PCNA (Proteintech Cat# 10205-2-AP, RRID:AB_2160330), and mouse anti-α-tubulin (1:2000) (Sigma-Aldrich Cat# T6199, RRID:AB_477583). Secondary antibodies used include goat anti-rabbit IgG-HRP (1:5000) (Abcam Cat# ab6721, RRID:AB_955447) and goat anti-mouse IgG-HRP (1:5000) (Thermo Fisher Scientific Cat# 62-6520, RRID:AB_2533947). Blots were processed using Pierce ECL Western Blotting Substrate kit (ThermoFisher Scientific Cat# 32109) and visualized with the ChemiDoc XRS+ Imaging system (RRID: SCR_019690) and image lab software (Bio-Rad Cat# 1709690, RRID:SCR_014210).

*Intracellular D-2-HG Quantification*

Intracellular D-2-HG was quantified using enzymatic assays originally described by Balss, *et al.* 2012 [1], and who we thank for generously providing key reagents that are also commercially available in the D-2-HG Assay Kit (Sigma-Aldrich Cat# MAK320). Briefly, cells were harvested and lysed prior to splitting each sample into two aliquots for D-2-HG quantification and total protein quantification using the Pierce BCA Protein Assay Kit (Thermo Fisher Scientific Cat# 23225), respectively. Deproteination of the D-2-HG quantification aliquot was achieved using 3 μL of Proteinase K (Qiagen Cat# 19131) per 100 μL of cell lysis solution. 25 μL of lysate was then added to 75 μL of assay solution containing 0.1 μg of the enzyme D-2-HG dehydrogenase (HGDH), 100 μM NAD+, 5.0 μM resazurin, and 0.01 U/mL diaphorase in 100 mM HEPES (pH 8.0). In the presence of D-2-HG, HGDH converts D-2-HG to α-ketoglutarate (α-KG) in a NAD+ dependent manner. The reduction of NAD+ to NADH enables the conversion of resazurin to fluorescent resorufin, which was then quantified via fluorometric detection (λ_ex_=540 nm, λ_em_=590 nm) on a Wallac Victor2 1420 Multilabel HTS Counter (Perkin Elmer Cat# 1420-041). D-2-HG quantification (pmole/μg protein) of a given sample was based on a standard curve of known D-2-HG concentrations. Two-tailed Student’s *t*-tests were used to compared means between groups, with statistical significance set at *P≤*0.05.

*Gene-Specific m6A RT-qPCR*

Gene-specific m6A RT-qPCR was performed by coupling m6A-immunoprecipitation and RT-PCR techniques. *Gaussia* luciferase (GLuc) m6A-positive control mRNA and *Cypridina* luciferase (CLuc) m6A-negative control mRNA were spiked into 10 μg of total RNA from samples. Immunoprecipitation of m6A-RNA was performed as described in the MeRIP-Seq protocol, but without any RNA fragmentation steps. The pulled down m6A-RNA was subjected to first-strand cDNA synthesis using a SuperScript II Reverse Transcriptase Kit with oligo-dT primers (Thermo Fisher Scientific Cat# 18064014). Briefly, 1 μL of Oligo(dT) was combined with 1 μL of 10 mM dNTP mix, 10 μL of m6A-immunoprecipitated RNA and sterile nuclease-free water for a final volume of 12 μL. The mixture was heated to 65 °C for 5 min and chilled on ice. 4 μL of 5X First-Strand Buffer, 2 μL of 0.1 M DTT, and 1 μL of RNaseOUT (Invitrogen Cat# 100000840) was then added to the mixture. The contents were mixed and then incubated at 25 °C for 2 min. 1 μL of SuperScript II RT was then added to the mixture to establish a final volume of 20 μL. The mixture was incubated at 25 °C for 10 min and then 42 °C for 50 min. The RT reaction was inactivated at 70 °C for 15 min. Next, 2 μL of the RT product was added to a PCR reaction mixture of 5 μL of 10X PCR Buffer (200 mM Tris-HCL (pH 8.4), 500 mM KCl), 1.5 μL of 50 mM MgCl_2_, 1 μL of 10 mM dNTP mix, 1 μL of forward primer (10 μM), 1 μL of reverse primer (10 μM), 0.4 μL of Taq DNA polymerase, and sterile nuclease-free water for a final volume of 50 μL. The mixture was then added in triplicate to a 96-well plate, and a denaturing step was performed by heating the mixture to 94 °C for 2 min. Real-time quantitative PCR data collection and analysis were performed on a Bio-Rad CFX96 Real-Time PCR Detection System (RRID:SCR_018064).

Primers utilized in these experiments include the following:

| Gene | Forward Primer | Reverse Primer |
| --- | --- | --- |
| *ACTB* | 5’- CCAGAGGCGTACAGGGATAG-3’ | 5’-CCAACCGCGAGAAGATGA-3’ |
| *ATF5* | 5’-GCTCAGAGGGAAGAGTGTCG-3’ | 5’-CAAGGCGAAAGTGGAAGACT-3’ |

*TCGA Gene Expression Analyses*

Gene expression data for 234 *IDH1^wt^* and 429 *IDH1^mut^* glioma samples were obtained from the GlioVis (RRID:SCR_023877) [2] database by downloading the Cancer Genome Atlas (TCGA) (RRID:SCR_003193) GBM+LGG RNA-Seq and phenotype datasets. RNA-Seq expression data were then cross referenced with patient phenotypes using custom scripts in R Project for Statistical Computing (RRID:SCR_001905) to sort patients by *IDH1* status. Normalized average expression for all transcripts, direction of expression (difference in average between *IDH1^wt^* and *IDH1^mut^*), and calculated probability statistics (Student’s *t*-test) were then generated for *IDH1^wt^* and *IDH1^mut^* patient samples for cross referencing with our MeRIP-Seq RADAR cell line data. *ATF5* survival analysis by expression was obtained through GlioVis by downloading the TCGA GBM Agilent dataset and separating into *IDH1^wt^* and *IDH1^mut^* patients.

*Gliomasphere Microarray Analyses*

Total RNA was isolated from gliomasphere cultures and subjected to poly A^+^ purification for mRNA. A Thermo Scientific Nanodrop 1000 Spectrophotometer (RRID:SCR_016517) was used to determine concentration and quality of RNA samples, followed by confirmatory assays using an Agilent 2100 Bioanalyzer Instrument (RRID:SCR_018043). Purified mRNA was then reverse transcribed, labeled, and prepared per the manufacturer’s protocols prior to hybridization to an Affymetrix high-density oligonucleotide HG-U133A Plus 2.0 Human Array to analyze gene expression levels for 47,400 transcripts/variants, including 38,500 genes (Affymetrix, RRID:SCR_010231). Microarray data deposited in Annotare; Accession number: E-MTAB-13765.

*Animal Procedures*

Female NOD SCID gamma (NSG) mice (RRID:IMSR_JAX:005557) (8-10 weeks old) were used for all experiments requiring intracranial xenografting of patient-derived glioma stem cell lines. Pharmacokinetic analysis of FB23-2 was performed as previously described [3]. Cell lines cultured in regular gliomasphere culture media were spun down at 300 g for 4 min and then disassociated using TrypLE Express Enzyme (1X, with phenol red) (Thermo Fisher Scientific Cat# 12605036) and gentle pipetting. Cell concentration calculations were performed on fractions resuspended to the single cell level, with a final concentration of 300,000 cells / 2 μL media prepared for each mouse and held on ice to await intracranial injection. NSG mice were placed into an isoflurane anesthesia induction chamber, and then affixed on a steady isoflurane flow via a nose cone coupled to a stereotactic stage. The incision area was shaved and sterilized with three rounds of betadine and ethanol scrubs. A 2 cm diagonal scalp incision was then made from the near the left eye posterior to the right rostrum. A stereotactic needle mount was then moved into position directly over the skull plate fusion landmark bregma where it was zeroed. The needle was then moved 1 mm anterior and 2 mm lateral (to the right) over the right basal ganglion injection site. A fine bit drill was then used to create a burr hole. The needle was then loaded with 300,000 gliomasphere cells suspended in a final injection volume of 2 μL. The stereotactic needle driver was zeroed upon the bevel reaching the surface of the brain within the burr hole. The needle was then lowered into the skull at a rate of 0.1 mm / 5 s until a depth of 2.5 mm was achieved. After 30 s, the needle was then raised at a rate of 0.1 mm / 5 s until reaching a final depth of 2.0 mm, thereby leaving behind a small cavity for tumor cell injection. The 2 μL tumor cell suspension was injected into the brain at a rate of 0.1 μL / 10 s. When finished, the needle was allowed to sit in the brain for 30 s before being drawn up at a rate of 0.1 mm / 10 s. The burr hole was then plugged with bone wax before closing the scalp incision with a tissue adhesive. Xenografted mice were then removed from the stereotactic head mount and isoflurane nose cone and transferred to a clean cage over a heating pad for individual monitoring until awake. Xenografted mice were allowed to recover for 3 d before being randomized into either FB23-2 or DMSO treatment groups. FB23-2 (MedChemExpress Cat# HY-127103) was dissolved in DMSO for a final stock concentration of 50 mM and was delivered via intraperitoneal (i.p.) injection (20 mg/kg) daily. Injection volumes were calculated each day based on individual mouse weights. DMSO control treatments were delivered in a similar fashion, with i.p. injection volumes scaling with animal weights. Mice were checked daily and euthanized by CO_2_ inhalation upon the display of symptoms indicative of excessive glioma tumor burden, including but not limited to, recurrent seizures, paralysis, hunched posture, decreased grooming and/or eating activity, and labored breathing. All treatment deliveries and censorship events were verified by two independent handlers. Overall survival (d) was calculated for each mouse at the time of censorship. While each group initially started with 10 mice, three XDS4130 mice were censored (FB23-2=2 mice, DMSO=1 mouse) due to death during GLuc measurements prior to their tumor burdens reaching moribund levels. Five GS187 mice were censored (FB23-2=3 mice, DMSO=2 mice) due to death during GLuc measurements (3 mice) or bowel perforation following daily intraperitoneal injections (2 mice) prior to their tumor burdens reaching moribund levels.

*FB23-2 Pharmacokinetic Studies*

As detailed in Tsang et al, 2020 [3], briefly, pharmacokinetic studies were performed on male CD-1 mice treated by intraperitoneal injection with 20mg/kg of FB23-2. Whole blood and brain tissue was collected following euthanization at t= 0, 0.25, 0.5, 1, 2, 4, 8, and 24 h post-treatment (*n*=2 mice per time point). Plasma was isolated via whole blood centrifugation, and brain tissue was homogenized in PBS. Centrifugation allowed for isolation of supernatant that was subsequently subjected to rotary evaporation and reconstitution in 100 μL 50:50:0.1 water:acetonitrile:formic acid. Chromatographic separations were performed using an Agilent 1290 Infinity LC system with a solvent flow rate of 0.10 mL/min. Mass spectrometry was performed using an Agilent 6460 triple quadrupole LC/MS system. Analyte signals were normalized to an internal standard and FB23-2 concentrations in each sample were calculated against a calibration curve.

*Gaussia Luciferase Tumor Burden Estimation*

Within the confinement of a laminar flow hood, mice were individually restrained and prepped with antiseptic isopropyl wipes for tail vein bleeding. The lateral tail vein was then lanced with a single-use steel lancet and 6.6 μL of venous blood were collected using a freshly tipped p10 pipet. Blood samples were promptly mixed with 2 μL of 50 mM EDTA to prevent coagulation and temporarily stored in PCR strips and on ice, but not for more than 2 h. Once all experimental and negative-control blood samples had been collected, an opaque 96-well plate was prepared. To each well, 8 μL of 5 mM NaCl in 1x PBS and 2 μL of blood were added, followed by homogenization. Blood samples were plated in triplicates, to allow for averaging of sample values. Separately, fresh substrate Coelenterazine (CTZ) (NanoLight Technology Cat# 303) was prepared and allowed to incubate in the dark at room temperature for 30 min. Substrate CTZ (100 μM) is a 1:100 dilution of 10 mM stock CTZ in salted PBS (5 mM). Enough substrate CTZ was produced to allow 100 μL to be added to each well containing sample in addition to 1.5 mL of substrate CTZ used for priming the luminometer injector. 10 mM CTZ aliquots were produced by adding 1.179 mL of acidified ethanol to 5 mg of stock CTZ. Acidified ethanol was produced by adding 2 drops of 1 M HCL to 10 mL of molecular grade ethanol. Stock CTZ was stored at -80 °C and away from light for long term storage. *Gaussia* luciferase readings were performed on a CLARIOstar luminometer and analyzed using MARS Data Analysis Software (RRID:SCR_021015). Before each use of the luminometer, the injector was rinsed twice with 70% ethanol and then primed with fresh substrate CTZ. Next, the luminometer was programmed to read luminescence following a flash kinetics model wherein readings are integrated over a 1 s interval. The 96-well plate containing samples was then loaded into the luminometer for program execution. Raw luminometer readings were multiplied by a coefficient of 5 to account for the 1:5 dilution of 2 μL of blood in 8 μL of salted PBS. *Gaussia* luciferase reporter assay readings were taken weekly for each mouse.

*Survival Analysis*

Overall survival data was collated in Prism 9. Comparisons of survival curves between groups were accomplished using log-rank (Mantel-Cox) tests and Gehan-Breslow-Wilcoxon tests, allowing for the calculation of *P* and Chi square values. 1 degree of freedom (df) was used in all survival analyses, and the threshold for significance was set at *P*≤0.05. Median survival times, log-rank Hazard Ratios, and 95% CI intervals were also calculated. Kaplan-Meier survival curves were generated using GraphPad Prism 9 (RRID:SCR_002798).

**REFERENCES**

1. Balss J. Enzymatic assay for quantitative analysis of (d)-2-hydroxyglutarate. *Acta Neuropathologica*. **2012**;124:883-891,.

2. Bowman RL, Wang Q, Carro A, Verhaak RGW, Squatrito M. GlioVis data portal for visualization and analysis of brain tumor expression datasets. *Neuro Oncol*. **2017**;19:139–41.

3. Tsang JE, Urner LM, Kim G, Chow K, Baufeld L, Faull K, et al. Development of a Potent Brain-Penetrant EGFR Tyrosine Kinase Inhibitor against Malignant Brain Tumors. *ACS Med Chem Lett*. American Chemical Society; **2020**;11:1799–809.
