# Supplementary material for: D-2-HG Inhibits IDH1mut Glioma Growth via FTO Inhibition and Resultant m6A Hypermethylation
Source: Cancer Res Commun. 2024 Mar 22;4(3):876–94. doi: 10.1158/2767-9764.CRC-23-0271 (PMC10959073; doi:10.1158/2767-9764.CRC-23-0271)
Supplement: Figure S1 — IDH1mut Production of D-2-HG Induces RNA m6A Hypermethylation. [file crc-23-0271-s04.pdf]

Supplementary Figure 1

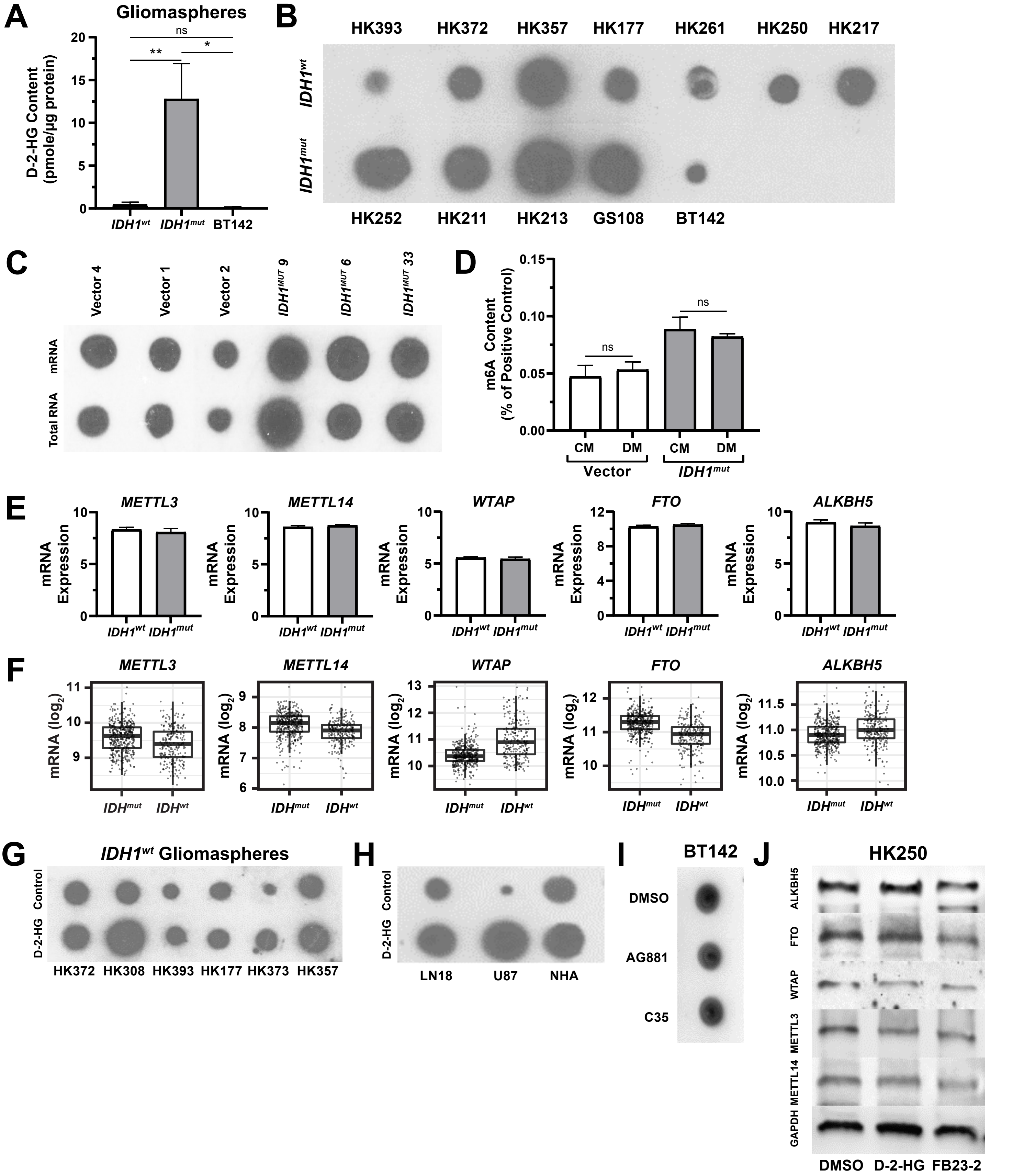

Supplementary Figure 1: *IDH1*^{mut} Production of D-2-HG Induces RNA m6A Hypermethylation. A: Intracellular D-2-HG content increased in heterozygous *IDH1*$^{mut/wt}$ gliomaspheres (HK252, HK211, HK213, GS108) compared to *IDH1*$^{wt/wt}$ gliomaspheres (HK393, HK372, HK385, HK296, HK261, HK250, HK217) and hemizygous *IDH1*$^{mut/-}$ BT142 gliomaspheres, measured via D-2-HG Enzymatic Assay. **B:** m6A content is visibly increased in heterozygous *IDH1*$^{mut/wt}$ gliomaspheres compared to *IDH1*$^{wt/wt}$ gliomaspheres and hemizygous *IDH1*$^{mut/-}$ BT142 gliomaspheres, detected via m6A dot blot. **C:** Vector and *IDH1*^{mut} forced expression in HEK293T cell lines exhibited similar m6A content in total RNAs and mRNAs measured by dot blot. **D:** Differentiation medium (DM) had no effect on m6A content in either vector or *IDH1*^{mut} forced expression in gliomaspheres (GS208 and GS243) when compared to complete medium (CM). **E:** Microarray expression patterns (mRNA) of m6A writers (*METTL3*, *METTL14* and *WTAP*) and erasers (*FTO* and *ALKBH5*) were similar between *IDH1*$^{mut/wt}$ and *IDH1*$^{wt/wt}$ gliomaspheres. **F:** Expression patterns (mRNA) of m6A writers and erasers in *IDH1*^{mut} and *IDH1*^{wt} patient glioma tissue samples obtained via TCGA (All p-values from the TCGA dataset are p<0.0001). **G** and **H:** Octyl-D-2-HG treatment induces increased m6A enrichment in *IDH1*$^{wt/wt}$ gliomaspheres (0.5 mM, E) and LN18, U87, and NHA (1.0 mM, F) cells via dot blot. **I:** Treatment with IDH1^{mut} inhibitors (AG881, = 1 μM, or C35, =2 μM) has no effect on intracellular m6A content in hemizygous *IDH1*$^{mut/-}$ BT142 gliomaspheres. **J:** Western blot images of m6A writers (METTL3, METTL14, and WTAP) and erasers (ALKBH5 and FTO) in *IDH1*^{wt} gliomaspheres (HK250) following DMSO, octyl-D-2-HG (0.5 mM), or FTO inhibitor (FB23-2, =3 μM) treatment. **P*≤0.05, ***P*≤0.01, ****P*≤0.001, and *****P*≤0.0001 compared to relevant controls. Unless otherwise stated, *P*-values indicate unpaired Student's *t*-test comparisons with the control, or between two groups as indicated by the horizontal line.
