# Supplementary material for: D-2-HG Inhibits IDH1mut Glioma Growth via FTO Inhibition and Resultant m6A Hypermethylation
Source: Cancer Res Commun. 2024 Mar 22;4(3):876–94. doi: 10.1158/2767-9764.CRC-23-0271 (PMC10959073; doi:10.1158/2767-9764.CRC-23-0271)
Supplement: Figure S2 — Effects of Octyl-D-2-HG and IDH1mut Inhibitors on Glioma Cell Growth. [file crc-23-0271-s05.pdf]

Supplementary Figure 2

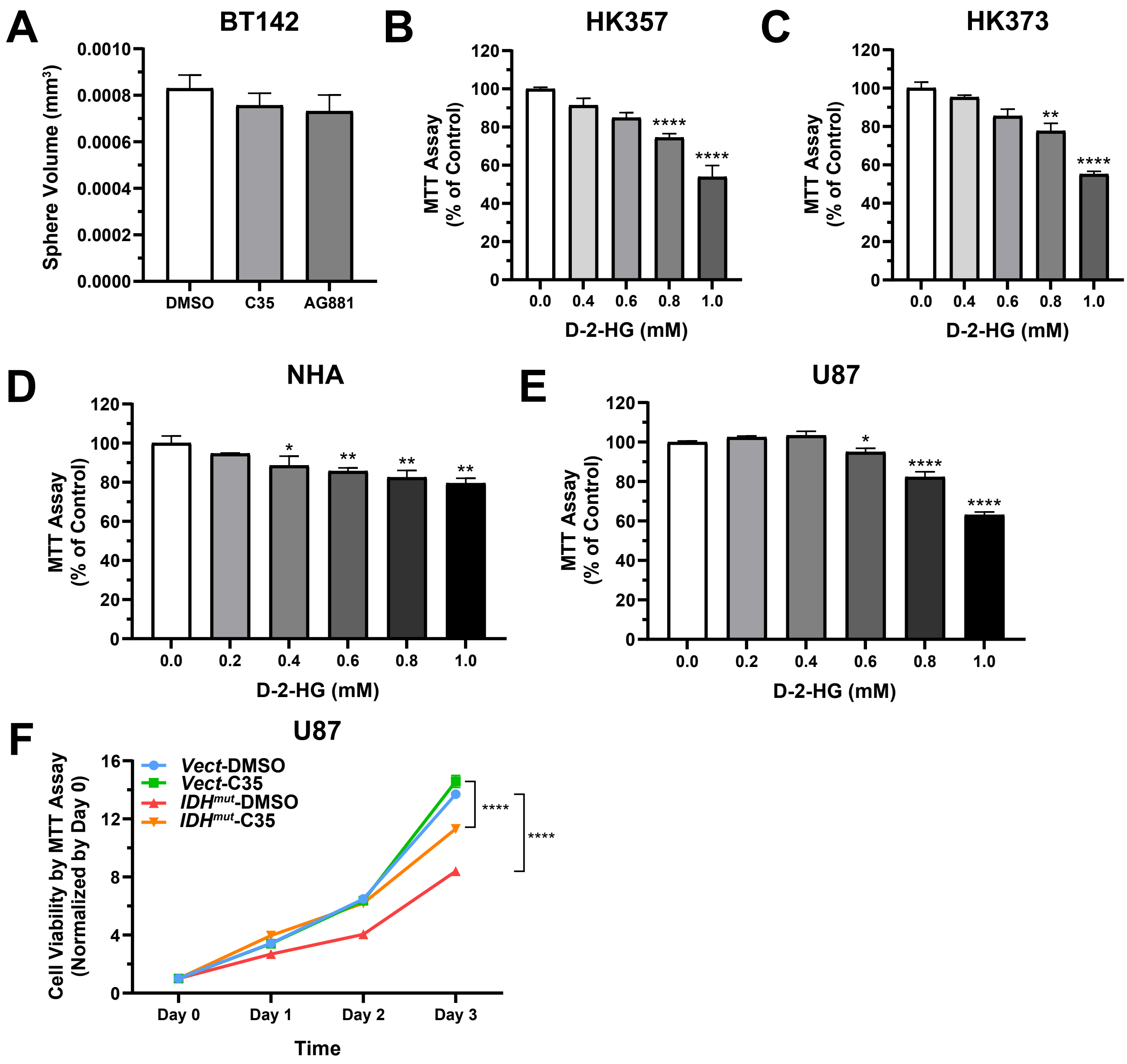

Supplementary Figure 2: Effects of Octyl-D-2-HG and IDH1^{mut} Inhibitors on Glioma Cell Growth. A: Hemizygous *IDH1*$^{mut/-}$ BT142 gliomaspheres are not sensitive to treatment with IDH1^{mut} inhibitors (AG881=1 μM; C35=2 μM)**. B**, **C**, **D** and **E:** Octyl-D-2-HG treatment (72 hours) demonstrated dose-dependent effects on cell viability in *IDH1*^{wt} gliomaspheres (HK357, B; HK373, C), NHA (D), and U87 (E) cell lines, measured via MTT assays. **F:** IDH1^{mut} inhibitor C35 (2 μM) treatment reversed attenuating effects on viability of *IDH1*^{mut} (mut clone #33, orange and red) forced expression in U87 cells (ANOVA, C35 treatment on cell viability at day 3: $F(1,12)=71.8$, $P \leq 0.0001$; *Vector* vs. *IDH*^{mut} at day 3: $F(1,12)=363.7$, $P \leq 0.0001$; interaction: $F(1,12)=20.7$, $P \leq 0.0007$). Asterisks indicate post-hoc Newman-Keuls comparisons at day 3 between groups indicated by the brackets. *$P \leq 0.05$, **$P \leq 0.01$, ***$P \leq 0.001$, and ****$P \leq 0.0001$ compared to relevant controls. Unless otherwise stated, *P*-values indicate unpaired Student's *t*-test comparisons with the control, or between two groups as indicated by the horizontal line.
