# Supplementary material for: D-2-HG Inhibits IDH1mut Glioma Growth via FTO Inhibition and Resultant m6A Hypermethylation
Source: Cancer Res Commun. 2024 Mar 22;4(3):876–94. doi: 10.1158/2767-9764.CRC-23-0271 (PMC10959073; doi:10.1158/2767-9764.CRC-23-0271)
Supplement: Figure S3 — Effects of FTO and ALKBH5 shRNA Knockdown or Overexpression on Glioma Cell Growth. [file crc-23-0271-s06.pdf]

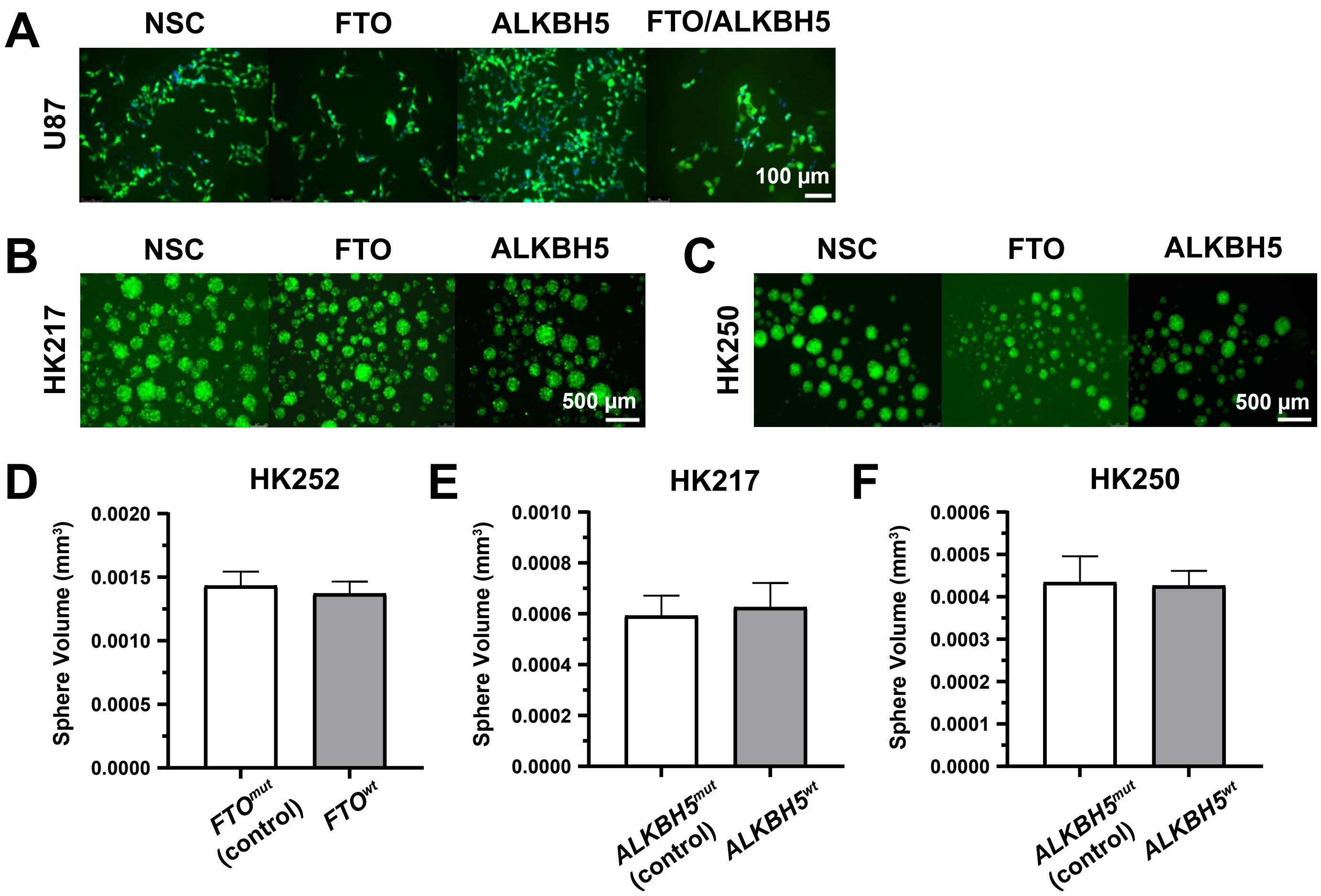

Supplementary Figure 3: Effects of *FTO* and *ALKBH5* shRNA Knockdown or Overexpression on Glioma Cell Growth. A, **B** and **C:** Fluorescent micrographs of U87 cells (20X magnification) and HK217 and HK250 gliomaspheres (10X magnification) following transfection of lentiviral vectors containing piLenti-*shRNA-EGFP-NSC* scrambled control, -*FTO* shRNA, or -*ALKBH5* shRNA. All U87 cells and gliomaspheres were GFP positive, and FTO knockdown reduced U87 cell proliferation and gliomasphere sphere size, while ALKBH5 knockdown had no obvious effects. **D:** FTO^{wt} overexpression compared to inactivated FTO^{mut} negative control had no measurable effect on sphere growth in $IDH1^{mut}$ gliomaspheres (HK252) at day 25 in culture. **E** and **F:** $ALKBH5^{wt}$ overexpression compared to inactivated $ALKBH5^{mut}$ negative control had no measurable effect on sphere growth in $IDH1^{wt}$ gliomaspheres (HK217, E; HK250, F) at day 9 in culture.
