# Supplementary material for: D-2-HG Inhibits IDH1mut Glioma Growth via FTO Inhibition and Resultant m6A Hypermethylation
Source: Cancer Res Commun. 2024 Mar 22;4(3):876–94. doi: 10.1158/2767-9764.CRC-23-0271 (PMC10959073; doi:10.1158/2767-9764.CRC-23-0271)
Supplement: Figure S4 — Pharmacologic Inhibition of FTO Results in Growth Inhibition of IDH1wt Gliomaspheres In Vitro. [file crc-23-0271-s07.pdf]

# *Supplementary Figure 4*

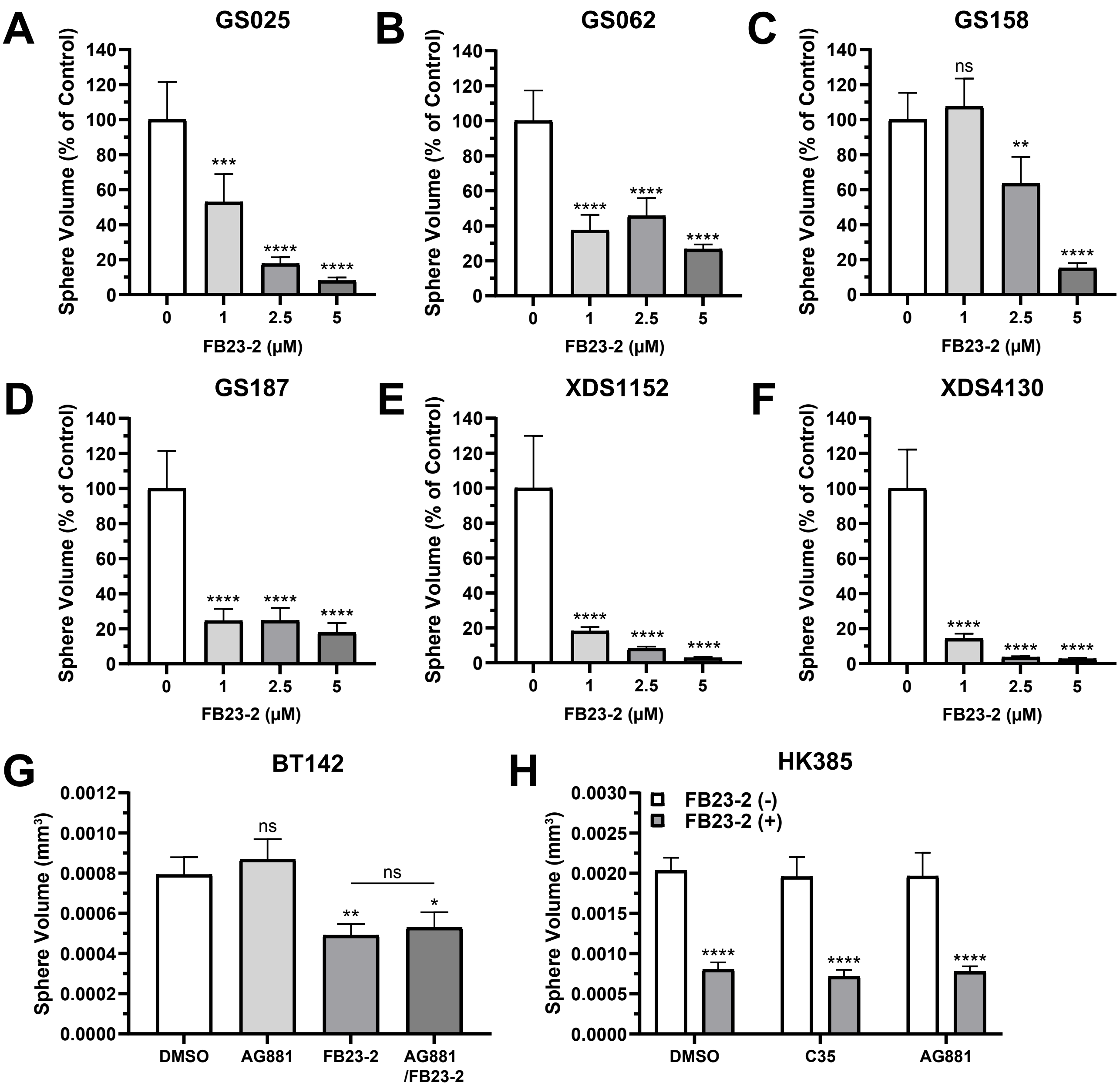

**Supplementary Figure 4: Pharmacologic Inhibition of FTO Results in Growth Inhibition of *IDH1$^{wt}$* Gliomaspheres *In Vitro*. A**, **B**, **C**, **D**, **E** and **F:** FTO-selective inhibitor FB23-2 demonstrated dose-dependent growth inhibiting effects on *IDH1$^{wt}$* gliomaspheres (GS025, A; GS062, B; GS158, C; GS187, D; XDS1152, E; and XDS4130, F). **G:** Effects of FTO inhibitor FB23-2 (3 μM), IDH1$^{mut}$ inhibitor AG881 (1 μM) or FB23-2+AG881 co-treatment on sphere growth in hemizygous *IDH1$^{mut/-}$* BT142 gliomaspheres. **H:** Effect of FTO inhibitor FB23-2 (3 μM) treatment on sphere growth in *IDH1$^{wt}$* HK385 gliomaspheres (ANOVA, FB23-2 treatment $F(1,1172)=78.6$, $P \leq 0.0001$; asterisks indicate post-hoc Newman-Keuls comparison for each treatment between with or without FB23-2). Treatment with C35 (2 μM) or AG881 (1 μM) were found to be similar to DMSO. *$P \leq 0.05$, **$P \leq 0.01$, ***$P \leq 0.001$, and ****$P \leq 0.0001$ compared to relevant controls. Unless otherwise stated, *P*-values indicate unpaired Student's *t*-test comparisons with the control, or between two groups as indicated by the horizontal line.
