# Supplementary material for: D-2-HG Inhibits IDH1mut Glioma Growth via FTO Inhibition and Resultant m6A Hypermethylation
Source: Cancer Res Commun. 2024 Mar 22;4(3):876–94. doi: 10.1158/2767-9764.CRC-23-0271 (PMC10959073; doi:10.1158/2767-9764.CRC-23-0271)
Supplement: Figure S5 — ATF5 is a Downstream Effector of the IDH1mut → D-2-HG ⊣ FTO Axis Resulting in Gliomasphere Growth Reduction. [file crc-23-0271-s08.pdf]

Supplementary Figure 5

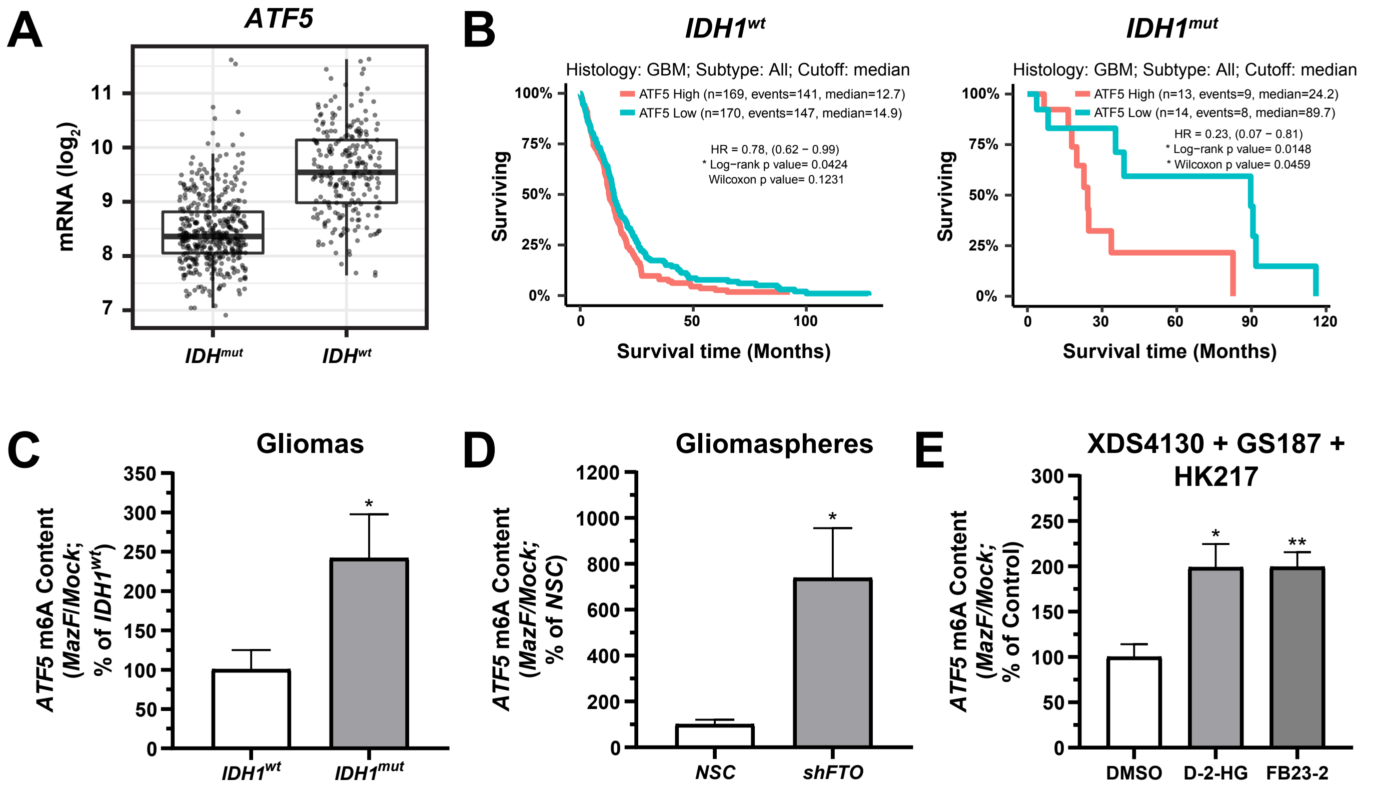

Supplementary Figure 5: ATF5 is a Downstream Effector of the *IDH1*^{mut} → D-2-HG ⊣ FTO Axis Resulting in Gliomasphere Growth Reduction. A: Reduced *ATF5* expression in *IDH1*^{mut} patient gliomas compared to *IDH1*^{wt} gliomas, assessed with GlioVis analysis of TCGA GBM+LGG RNA-Seq data (P<6.6E-65, pairwise t-test with Bonferroni multiple testing correction). **B:** *ATF5* expression is inversely associated with overall survival in high-grade glioma (TCGA GBM), assessed with GlioVis analysis of TCGA survival data for *IDH1*^{wt} and *IDH1*^{mut} patients. **C:** MazF-*ATF5* RT-qPCR data providing orthogonal validation of *ATF5* enrichment in *IDH1*^{mut} (n=19) vs *IDH1*^{wt} (n=14) gliomas obtained from patient tissue (P≤0.03). **D:** MazF-*ATF5* RT-qPCR showing m6A enrichment in *ATF5* mRNA following FTO shRNA knockdown in *IDH1*^{wt} (HK217, HK250) gliomaspheres, compared to NSC-shRNA control (P≤0.02). **E:** MazF-ATF5 RT-qPCR pooled data from *IDH1*^{wt} (XDS4130, GS187, HK217) gliomaspheres showing induction of ATF5 mRNA m6A enrichment following octyl-D-2-HG (0.5 mM) or FB23-2 (3 μM) mediated FTO inhibition. *P≤0.05, **P≤0.01, ***P≤0.001, and ****P≤0.0001 compared to relevant controls. P-values indicate unpaired Student's t-test comparisons with the control, or between two groups as indicated by the horizontal line.
